# Performance enhancement of a sugarcane bagasse-fired steam power plant through flue gas-assisted drying: a case study of Metehara Sugar Factory, Ethiopia

**DOI:** 10.1038/s41598-026-50724-7

**Published:** 2026-04-25

**Authors:** Haftamu Kahsay G/Micheal, Gabr Goshu Syum

**Affiliations:** 1 Faculty of Chemical Engineering, EiT-M, , Mekelle University, P.O. Box 231, Zip Code: 7000 Mekelle, Tigray Ethiopia; 2 Faculty of Mechanical and Industrial Engineering, EiT-M, Mekelle University, P.O. Box 231, Zip Code: 7000 Mekelle, Tigray Ethiopia; 3 Centre of Excellence in Studies in Oil and Gas Engineering and Technology (CS-OGET), Eduardo Mondlane University, Av. de Moçambique, Km 1.5, Bairro Luís Cabral, Maputo 257, Mozambique

**Keywords:** Cogeneration system, Moisture reduction technique, Steam cycle performance, Waste-to-energy utilization, Industrial case study, Ethiopia, Energy science and technology, Engineering

## Abstract

This study investigates the performance enhancement of a sugarcane bagasse-fired steam power plant at the Metehara Sugar Factory in Ethiopia by integrating flue-gas-assisted biomass drying. The primary objective is to evaluate how reducing the high as-received moisture content (~ 46%) of bagasse improves fuel quality and overall plant performance. A thermodynamic model of the existing simple Rankine cycle was developed using Engineering Equation Solver (EES) with IAPWS-IF97 water–steam properties. The model was applied to assess plant performance under two operating conditions: a base case using as-received bagasse and an upgraded case incorporating flue gas-assisted drying. Key operating parameters, including turbine inlet pressure (2 MPa) and condenser pressure (95 kPa), were maintained constant, while turbine inlet temperature varied based on combustion conditions. In the base case, the plant generates 9.03 MW of net power with a thermal efficiency of 22.56%. With the integration of flue gas-assisted drying, the reduction in moisture content increases the lower heating value and combustion temperature, leading to improved steam conditions. As a result, net power output increases to 16.61 MW, and thermal efficiency improves to 27.35%, representing an efficiency gain of 4.8 percentage points without additional fuel input. The results demonstrate that utilizing waste heat from boiler exhaust gases for biomass drying significantly enhances energy recovery and power generation capacity. This approach provides a practical and low-cost solution for improving the performance of biomass-fired power plants and supports more efficient utilization of available energy resources in the sugar industry.

**Supplementary Information:**

The online version contains supplementary material available at 10.1038/s41598-026-50724-7.

## Introduction

Biomass-derived resources are increasingly regarded as key contributors to the global transition toward sustainable, low-carbon energy systems. Various studies have demonstrated the effective transformation of biomass waste into porous carbon through innovative synthesis methods, leading to materials with high surface areas and excellent capacitance^1^. In contrast to fossil fuels, whose carbon emissions originate from geological carbon stocks, biomass is considered largely carbon–neutral because the carbon dioxide released during combustion is approximately balanced by the amount absorbed during plant growth. This life-cycle attribute, coupled with the biodegradability and renewable nature of biomass-based materials, positions biomass as a strategic component of future clean-energy portfolios. Recent studies highlight that biomass-derived feedstock’s not only serve as energy resources, but are also gaining attention in advanced research areas, including bio-composites, functional polymers, biosensors, electrochemical and storage systems, demonstrating their wide-ranging potential to substitute conventional fossil-dominated materials and support circular-economy pathways^2^. Globally, biomass ranks as the fourth largest energy source after coal, oil, and natural gas, supplying roughly 14% of the world’s primary energy^3^. In many developing countries, especially in Sub-Saharan Africa, the share is considerably higher, with up to 90% of energy needs met by biomass, most of it used in traditional forms for cooking and heating.

The role of biomass in electricity generation has grown steadily over the past two decades, driven by technological advances and the urgent need to diversify energy systems^4^. However, its full potential remains underexploited in many regions due to technical, economic, and logistical challenges. One of the most critical technical barriers is the high moisture content of many biomass types, which reduces their lower heating value (LHV), lowers furnace temperatures, and decreases thermal efficiency in power generation systems. Moisture content varies widely depending on the biomass type and source, from less than 10% for cereal straw to over 60% for forestry residues, sewage sludge, and fresh algae^5^. Although biomass with 55–65% moisture can sustain combustion, high-moisture fuels are not ideal for direct firing without pre-treatment, as significant energy is consumed in evaporating water before effective furnace can occur^6^.

In industrial applications, such as sugarcane bagasse-fired steam power plants, high moisture content presents additional challenges. Sugarcane bagasse, the fibrous residue from sugarcane milling, is a key biomass resource in sugar-producing countries. It typically contains 45–50% moisture as it leaves the milling process. This moisture significantly lowers its LHV, meaning more fuel must be burned to achieve the same thermal output. Moreover, the extra water vapor in the flue gas increases heat losses through the boiler stack, reduces flame temperature, and can exacerbate corrosion and fouling in the boiler and downstream equipment.

Pre-drying biomass before the furnace is a well-established strategy for overcoming these limitations^7^. Drying increases, the LHV, raises furnace temperatures, enhances boiler efficiency, and reduces pollutant emissions. However, conventional biomass drying can be energy-intensive, requiring external heat sources that may erode the net efficiency gains if not carefully managed^8^. A more sustainable approach is to use low-grade waste heat, such as flue gas from the furnace process, for drying^9^. Flue gas-assisted drying offers a cost-effective solution that recycles energy that would otherwise be lost to the environment, thereby improving overall plant efficiency without increasing fuel consumption^10^.

In the Ethiopian context, the sugar industry represents a particularly promising opportunity for implementing such improvements. Ethiopia is expanding its sugar production capacity, with several operational factories and more under development. Sugarcane bagasse is generated in large quantities at these facilities, and while some is used for process heat or cogeneration, significant potential remains for electricity production^11^. The Metehara Sugar Factory, located in the Oromia region approximately 200 km southeast of Addis Ababa, is one of the country’s oldest and most established sugar producers. Processing over one million tons of sugarcane annually, it generates more than 170,000 tons of sugarcane bagasse each year, a resource that, if fully utilized, could significantly enhance the factory’s energy self-sufficiency and even enable surplus electricity export to the national grid^12^.

Despite this potential, Metehara’s current sugarcane bagasse-fired steam power system operates without integrated fuel pre-drying. This limits its thermal efficiency and power output, constrains its ability to meet internal electricity needs, and increases reliance on the national grid. Moreover, there has been limited research in Ethiopia and the wider region on the technical benefits of flue gas-assisted drying for biomass-fired plants, particularly using site-specific performance modelling and simulation tools^13^.

This study addresses this gap by assessing the technical feasibility and performance gains of integrating a flue gas-assisted sugarcane bagasse drying system into the Metehara Sugar Factory’s steam power plant. The significance or importance of this study lies in its demonstration of how an abundant, locally available biomass resource sugarcane bagasse can be more efficiently converted into electricity through the strategic recovery of waste heat within an existing industrial power system^14^. While sugarcane bagasse is widely used as a boiler fuel in sugar industries, its high as-received moisture content severely limits furnace temperature, steam quality, and overall power plant efficiency^15^. Most existing bagasse-fired power plants in developing countries operate without integrated fuel pre-treatment, resulting in underutilization of biomass energy potential and increased reliance on grid electricity or auxiliary fuels. In this context, the present study makes a novel contribution by introducing and quantitatively evaluating flue gas-assisted drying as an in-situ, low-cost efficiency enhancement strategy, using a site-specific industrial case study from Ethiopia^16^.

The novelty of this work lies in its application of site-specific performance modelling to a real industrial facility in Ethiopia, demonstrating how drying-enhanced biomass, viewed as both a renewable material and a thermodynamic resource, can substantially improve power output and cycle efficiency^17^. Furthermore, this research contributes to emerging knowledge on biomass upgrading for industrial energy applications, providing evidence that waste-heat recovery can elevate low-grade agricultural residues into high-value clean-energy inputs capable of supporting regional electricity stability. Unlike previous studies that focus on generic biomass systems or isolated drying analyses, this research integrates fuel characterisation, furnace analysis, waste heat recovery, and power cycle simulation into a single framework^18^. The results demonstrate that recovering waste heat from boiler exhaust gases to pre-dry sugarcane bagasse can increase net power output from 9.03 to 16.61 MW and improve thermal efficiency from 22.56 to 27.35% without additional fuel input^19^. These findings highlight the strong potential of biomass-derived materials, when properly conditioned, to serve as reliable and efficient energy carriers for industrial cogeneration, rural electrification, and sustainable energy development^20^. The proposed approach is technically replicable and highly relevant to sugar industries and agro-industrial facilities across Ethiopia and other biomass-rich regions worldwide.

- **Statement of research objectives**.

The proposed flue gas–assisted drying system is designed to achieve the following three primary objectives. Those are: (1) To assess the availability and characteristics of sugarcane bagasse at Metehara Sugar Factory with a **s**ite-specific focus on Metehara Sugar Factory, (2) A thermodynamic modelling and simulation of the existing simple Rankine cycle**,** representing current plant operation, and (3) To integrate and evaluate the impact of flue gas-assisted drying on plant thermal efficiency, power output, and operational performance. By focusing on a real industrial case in Ethiopia, the research provides evidence that can inform both plant-level decision-making and national energy policy, offering insights into how existing biomass resources can be more effectively harnessed for clean, reliable, and economically viable electricity generation^21^.

## Materials and methods

### Study area and site description of Metehara Sugar Factory

**Fig. 1:**
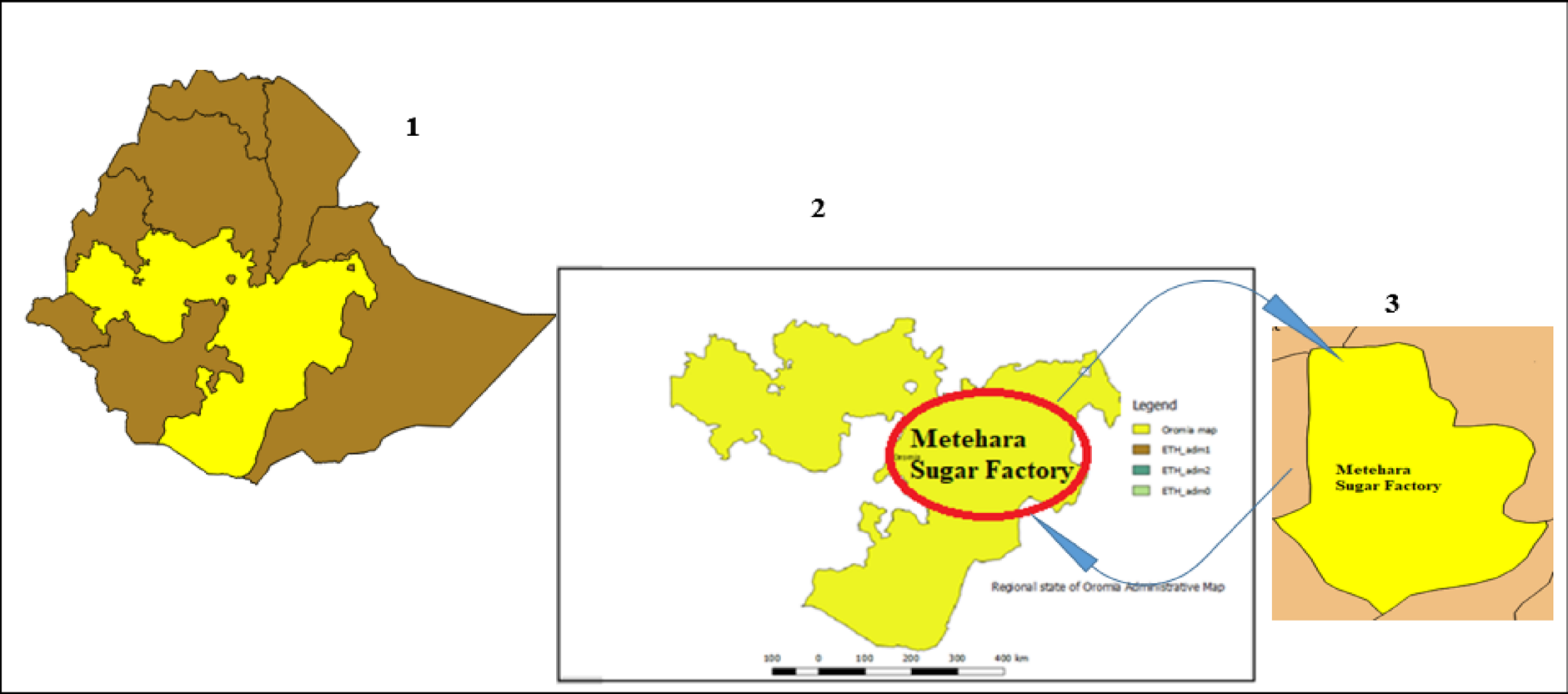
Locational map of Metehara Sugar Factory, Ethiopia. The map was modelled by the authors using QGIS version 2.18.0 (QGIS Development Team, 2016), an open-source geographic information system available at URL: https://diva-gis.org/index.html and https://qgis.org. Administrative boundary shape files for Ethiopia and all other regional states were obtained from publicly available open-source geographic datasets and created for visualization.

The Metehara Sugar Factory is one of the oldest and most established sugar production facilities in Ethiopia, located in the Oromia Regional State within the Upper Awash Valley, approximately 200 kms southeast of Addis Ababa along the Addis Ababa–Dire Dawa–Djibouti highway^22^. The Metehara Sugar Factory is located in the Upper Awash Valley of Ethiopia within the Oromia Regional State” (Ethiopian Sugar Corporation, 2023)^23^. Strategically positioned near major transportation corridors, the factory has direct logistical access for both domestic distribution and potential export of sugar and related by-products^24^. The surrounding region offers favourable climatic and soil conditions for sugarcane cultivation, with relatively stable year-round temperatures and access to irrigation water from the Awash River^25^. Figure 1. Shows the Locational map of Metehara Sugar Factory. This map was modelled using the QGIS version 2.18.0 Open Access software. In the locational map, the designations 1, 2, and 3 indicated for the Ethiopian map, Oromia regional state, and the specific location of the Metehara sugar factory, respectively as shown in Fig. 1.

The Metehara Sugar Factory, commissioned in 1965 as a joint venture between the Ethiopian government and the Dutch construction company Handels Vereeniging Amsterdam (HVA), was fully nationalised in 1975^26^. Over decades of operation, it has become a key player in Ethiopia’s agro-industrial sector, contributing significantly to rural employment, foreign exchange earnings, and the national sugar supply^27^. The factory manages approximately 10,230 hectares of sugarcane plantation, supported by a modern irrigation system, and operates a cane crushing capacity of 5,000 tons per day^28^. At full operational capacity, Metehara produces around 136,692 tons of refined sugar annually^29^.

Sugarcane processing at Metehara generates substantial quantities of sugarcane bagasse, the fibrous residue remaining after juice extraction. Based on the plant’s production data, the annual quantity of sugarcane bagasse generated is approximately 170,820 tons available for energy generation after accounting for contingency reserves^30^. The sugarcane bagasse is currently used mainly as boiler fuel for process steam generation, but without integrated fuel pre-drying, its high moisture content (typically 45–50% as received) limits the thermal efficiency of the furnace process^31^.

The factory’s energy infrastructure is based on a biomass-fired steam power plant employing a simple Rankine cycle configuration^32^. Steam is generated in high-pressure boilers using sugarcane bagasse as the primary fuel and is directed to turbines for electricity production, with the exhaust steam used for various process heating needs in sugar production^33^. While this cogeneration setup enables partial self-sufficiency, current plant performance leaves room for improvement, especially in meeting peak electrical loads and reducing reliance on the national grid^34^.

In addition to its operational importance, Metehara’s long history, established supply chain, and substantial biomass resource make it an ideal case study for evaluating efficiency-enhancing measures^35^. The integration of flue gas-assisted drying, using waste heat from boiler exhaust gases to reduce sugarcane bagasse moisture content before combustion, has the potential to improve thermal performance, increase net power output, and enhance environmental sustainability^36^. The site’s consistent and predictable sugarcane bagasse supply, combined with the availability of waste heat streams, provides the necessary conditions for implementing and testing such an upgrade^37^.

### Fuel sampling and characterization

Representative sugarcane bagasse samples were collected from the conveyor system immediately after juice extraction to capture typical as-received conditions. To ensure accuracy, sampling followed a composite approach, mixing multiple sub-samples collected at different times during daily operations^38^.

**Proximate analysis** was performed at the Messebo Cement Factory laboratory to determine:

- Moisture content (% by weight)
- Volatile matter content (%)
- Fixed carbon content (%)
- Ash content (%)

**Ultimate analysis** was conducted using reference data from the Energy Research Centre of the Netherlands (ECN) Phyllis biomass database, validated against laboratory ash content measurements^39^. This provided elemental composition values for:

- Carbon (C)
- Hydrogen (H)
- Oxygen (O)
- Nitrogen (N)
- Sulphur (S)

The Higher Heating Value (HHV) was measured using an adiabatic bomb calorimeter under standard laboratory conditions^40^. The Lower Heating Value (LHV) was calculated from the HHV using the hydrogen content and moisture data, applying established fuel energy equations. The relationship between LHV and moisture content was then plotted to assess the impact of drying on fuel energy potential^41^.

### Determination of furnace flame temperature

The maximum adiabatic flame temperature (**T**_**f**_) for the sugarcane bagasse furnace was estimated using a steady-state energy balance applied to the boiler furnace. The method accounted for the heat release from fuel combustion, the enthalpy of reactants and products, and the heat required to evaporate inherent moisture^42^.

Calculations were performed using MS Excel Solver to iteratively satisfy the energy balance equation under stoichiometric air–fuel conditions. The resulting flame temperature was determined for both as-received and dried sugarcane bagasse conditions, allowing quantification of the performance benefit from moisture reduction^43^.

### Power plant modelling and simulation

The steam power plant at Metehara was modelled as a simple Rankine cycle, consistent with its current operational setup^44^. The model incorporated the following major components:

- Biomass-fired boiler (steam generator)
- Steam turbine
- Condenser
- Feed water pump

Thermodynamic properties of water and steam were obtained using the IAPWS-IF97 formulation via the Engineering Equation Solver (EES) software with the Water-97 add-in. The cycle was modelled for two scenarios^45^:

1. **Base case:** As-received sugarcane bagasse (approximately 46% moisture) without drying
2. **Upgraded case:** Pre-dried sugarcane bagasse using flue gas-assisted drying to reduce moisture content before combustion

In each scenario, turbine inlet pressure and condenser pressure were held constant at 2 MPa and 95 kPa, respectively, while turbine inlet temperature was varied according to achievable furnace conditions^46^.

### Flue gas-assisted drying integration

The flue gas drying concept was modelled as a heat exchanger between the boiler exhaust gas and the incoming sugarcane bagasse stream. Key modelling assumptions included^47^:

- Steady-state operation
- Negligible heat loss to the environment
- No chemical interaction between flue gas and sugarcane bagasse
- Moisture reduction to an optimal level for the furnace without risk of excessive dryness (which could cause handling issues)

The waste heat available from the flue gas was estimated based on measured exhaust gas temperature and flow rate, adjusted for boiler load conditions. The recovered heat was used to calculate the achievable moisture reduction and the corresponding increase in LHV^48^.

### Adiabatic flame temperature calculation using MS excel solver

The following three basic points are necessary to calculate the adiabatic flame temperature of the furnace and the proposed system**.** Those are**:** governing equations, input assumptions, and MS Excel solver setup.

**(a). Governing Equation**—the adiabatic flame temperature (T_f_) was determined based on a steady-state energy balance applied to the combustion chamber under adiabatic conditions. The governing equation used in the model is:

$${\mathrm{H}}_{{\mathrm{R}}} \, + \,{\mathrm{LHV}}\, - \,{\mathrm{H}}_{{\mathrm{P}}} \, = \,0,$$

Where:

- H_R_ = total enthalpy of reactants (fuel + air)
- LHV = lower heating value of sugarcane bagasse (function of moisture content)
- H_P_ = total enthalpy of combustion products at flame temperature T_f_. This equation ensures that the total energy released during combustion equals the sensible enthalpy of the combustion products at the adiabatic flame temperature.

**(b) Input Assumptions—**the following some assumptions were used in the calculation:

- Steady-state and adiabatic combustion (no heat loss to surroundings)
- Complete combustion of sugarcane bagasse
- Stoichiometric air–fuel ratio (no excess air considered)
- Reference temperature: 25 °C for reactants
- Fuel composition: obtained from ultimate analysis (C, H, O, N, S)
- Moisture content effect included, accounting for:
- Combustion products considered: CO₂, H₂O (vapor), N₂

- Reduction in LHV
- Energy required for moisture evaporation

- Combustion products considered: CO₂, H₂O (vapor), N₂

**(c) MS Excel Solver Setup—**the adiabatic flame temperature was obtained numerically using MS Excel Solver with the following configuration:

**Decision variable—**Flame temperature (T_f_ ​).

**Objective function—**Energy balance equation.

$${\mathrm{f}}\left( {{\mathrm{T}}_{{\mathrm{f}}} } \right)\, = \,{\mathrm{H}}_{{\mathrm{R}}} \, + \,{\mathrm{LHV}}\, - \,{\mathrm{H}}_{{\mathrm{P}}} .$$

**Solver goal—**Set f(T_f_) = 0.

Method used**:** Iterative nonlinear solving (GRG Nonlinear method). The Solver iteratively adjusts (T_f_) until the energy balance residual approaches zero, ensuring convergence to the correct adiabatic flame temperature.

### Performance analysis of parameters

Cycle efficiency, net power output, and steam mass flow rate were calculated for each case using standard thermodynamic equations for open systems, applying the steady-flow energy equation. Energy and mass balances were used to determine^49^. Table 1 indicates the performance parameters and governing equations of the system.

**Table 1:** Performance parameters and governing equations.

Performance parameters	Governing formulas	Equation
Boiler heat input, Qin in (KJ/Kg)	Qin = h_3_ – h_2_	En.(1)
Turbine work output, W_t, output_ in (KJ/Kg)	W_t, output_ = h_3_ – h_4_	En. (2)
Pump work input, W_p, in_ (KJ/Kg	W_p, in_ = h_2_ – h_1_ = v (P_2_-P_1_)	En.(3)
Condenser heat rejection, Q_out_ in (KJ/Kg)	Q_out_ = ∆H = Q_out_ = h_4_—h_1_	En.(4)
Network output, W_net_ in (KJ/Kg)	W_turbine_, _out_ -W_pump_, _in_ = Q_in_—Q_out_	En.(5)
Mass flow rate of steam, in (Kg/Sec)	(mst) = $\frac{{{\mathrm{HP}}}}{{\text{h3 - h2}}}$	En. (6)
Energy balance relationship	HP = ṁst ∗ [h3 − h2]	En. (7)
Thermal efficiency, in (η_th_)	Ƞ_thermal_ = $\frac{{{\mathrm{Wnet}}}}{{{\mathrm{Qin}}}}$	En. (8)

The performance parameters presented in Table 1 also provide qualitative insight into the environmental behaviour of the system. An increase in boiler heat input (Qin) due to improved fuel quality enhances combustion efficiency, leading to more complete fuel utilization and reduced unburned emissions. Higher turbine work output (Wt) and network output (Wnet) indicate improved energy conversion efficiency, meaning more electricity is generated per unit of fuel, thereby lowering emissions intensity. Similarly, a reduction in condenser heat rejection (Qout) reflects decreased energy losses to the environment, which contributes to overall system efficiency. The increase in thermal efficiency (ηth) demonstrates that a larger fraction of the input energy is converted into useful work, reducing waste heat and associated environmental impacts. Furthermore, improved steam mass flow rate and energy balance relationships ensure stable and efficient operation, minimizing inefficiencies that could lead to excess fuel consumption and emissions. Overall, these performance improvements collectively contribute to reduced heat losses and lower environmental impact of the power generation system.

The values of enthalpies (h_2_ & h_3_) were calculated using the water97 add-in software tool at their corresponding temperature (K) and pressure (bar).

Where, W_net_ = W_t, out −_ W_p, in_ = Q_in_ − Q_out_ = (h_3_ – h_2_) − (h_4_ – h_1_).

Performance improvements were expressed as percentage changes relative to the base case. Graphical analyses were conducted to illustrate the influence of moisture content on flame temperature, steam conditions, and plant efficiency^50^.

### Summary of methodological workflow

**Fig. 2:**
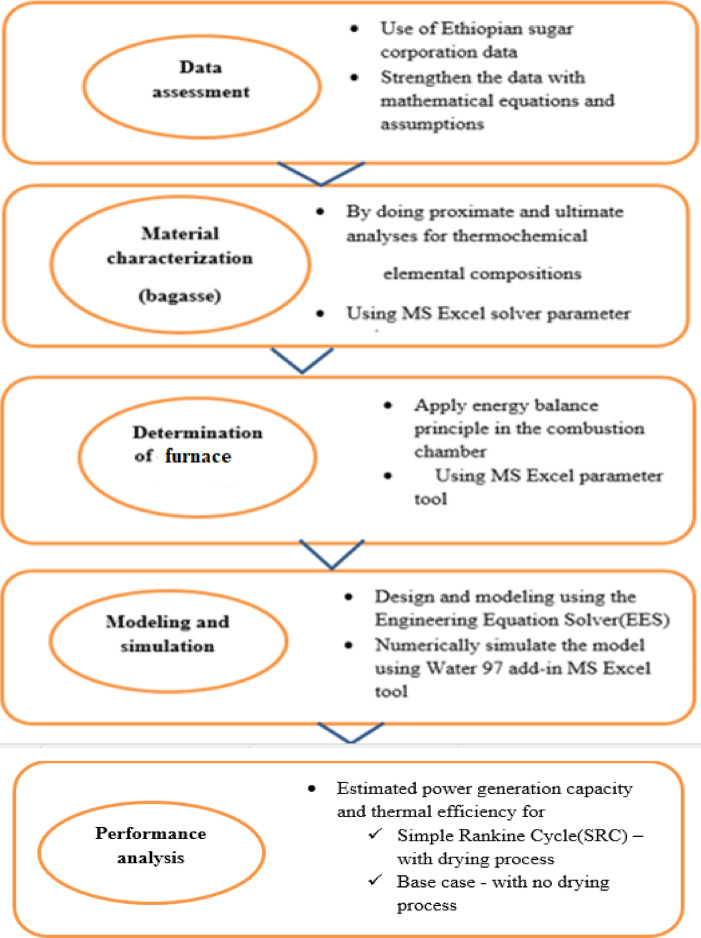
Overall flow chart of the methods.

The overall methodology followed five main steps: data assessment, material characterizations, determination of boiler furnace flame temperature, modelling and simulation of a furnace-based biomass power plant, and performance analysis, as illustrated in Fig. 2.

This structured approach ensured that each stage of the analysis built directly on site-specific empirical data, enhancing the reliability and applicability of the results^51^.

### Software and computational tools

Thermodynamic modelling of the Rankine cycle was performed using Engineering Equation Solver (EES) version 10.757 (F-Chart Software, USA), available at URL: https://fchartsoftware.com/ees/. Water and steam thermodynamic properties were obtained using the IAPWS-IF97 formulation through the Water97 add-in integrated within Engineering Equation Solver (EES) version 10.757 (F-Chart Software, USA), available at https://fchartsoftware.com/ees/. Graphical analysis and numerical iteration for adiabatic flame temperature calculations were carried out using Microsoft Excel 2016 (Microsoft Corporation, USA), using the built-in Solver tool available at https://www.microsoft.com/microsoft-365/excel. The locational map of Metehara Sugar Factory, Ethiopia (Fig. 1) was developed and modelled using QGIS version 2.18.0, an open-source geographic information system available at URL: https://diva-gis.org/index.html and https://qgis.org.

## Results and discussion

### Sugarcane bagasse resource availability at Metethara Sugar Factory

The availability of biomass feedstock is a fundamental determinant of the technical and economic viability of biomass-based power generation systems. For this study, the primary feedstock is sugarcane bagasse, the fibrous residue left after juice extraction during sugar processing. Sugarcane bagasse typically contains a mixture of cellulose, hemicellulose, lignin, and residual sugars, with a high inherent moisture content. In the Ethiopian sugar industry, sugarcane bagasse is often underutilized, with significant quantities either stockpiled or burned inefficiently for low-grade process heat.

At Metehara Sugar Factory, annual cane production and processing statistics form the basis of the resource assessment. Factory records indicate that the mill processes approximately 1,825,000 tons/year of harvested cane, of which about 1,095,000 tons/year are classified as millable sugarcane stalks, representing 60% of the total crop yield. The residue-to-product ratio (RPR) for sugarcane, defined as the mass of sugarcane bagasse produced per unit mass of processed cane, is typically in the range of 0.22–0.30 for well-optimized mills. In Metehara’s case, an RPR of 0.24 was observed, yielding a total of 262,800 tons/year of sugarcane bagasse as shown in Table 2^52^.

**Table 2:** Assessed the result of the summary of waste biomass (sugarcane bagasse) (52).

Sugarcane bagasse assessed parameters	Results	Unit
Total amount of mill able sugar cane stalks produced	1,095,000	ton/year
Total amount of residue produced	262,800	ton/year
Residues used for contingency	91,980	ton/year
Biomass residue available for energy production	170,820	ton/year

Not all of this sugarcane bagasse is available for electricity generation. Approximately 35% (91,980 tons/year) is retained for contingency purposes, including boiler start up, drying auxiliary fuel, and operational reliability during low-yield periods. After accounting for these internal allocations, the net annual sugarcane bagasse availability for power generation is 170,820 tons/year, corresponding to a continuous feed rate of 5.42 kg/s.

This level of resource availability is significant for several reasons:

- *Energy potential*—At the measured Higher Heating Value (HHV) of 15,990 kJ/kg (dry basis), this mass flow could theoretically provide a continuous thermal energy input of ~ 86.7 MW, depending on moisture content.
- *Capacity matching*—Given that the power plant under study is small-scale (< 20 MW), the available sugarcane bagasse resource is sufficient to operate at or near full capacity year-round, assuming effective storage and handling systems.
- *Reliability of supply*—Unlike seasonal residues from crops such as maize or wheat, sugarcane bagasse production aligns with the mill’s crushing season. In Metehara, the crushing season spans most of the year, ensuring a relatively stable biomass feed supply.
- *Potential for surplus power*—If the plant’s electrical demand is met, surplus electricity could be exported to the national grid, supporting rural electrification targets.

However, the inherent variability of sugarcane bagasse quantity and quality should be noted. Variations in cane yield, sucrose content, and processing efficiency can affect annual and seasonal feedstock availability. Furthermore, during maintenance shutdowns or off-season periods, sugarcane bagasse production may fall to zero, necessitating either storage solutions or supplementary fuels.

In summary, the sugarcane bagasse resource assessment confirms that the Metehara Sugar Factory has sufficient biomass feedstock to support a continuous small-scale power generation system. This finding provides the foundation for subsequent analysis of how pre-drying and flue gas heat recovery can maximize the conversion efficiency of this resource into electricity.

### Fuel characterization

Characteristics: The energy performance of a biomass-fired steam power plant depends heavily on the quality and composition of the fuel. Sugarcane bagasse’s thermochemical properties determine its furnace behaviour, flame temperature, and ultimately the steam parameters delivered to the turbine. **Proximate analysis** of Metehara’s sugarcane bagasse sample, carried out using a bomb calorimeter and standard fuel testing methods, are summarized in Table 3.

**Table 3:** Summary of proximate analysis result of waste biomass fuel (sugarcane bagasse).

Characterized Parameters	Results	Units
Heating values	15,990.243 14,780.179	KJ/kg
Moisture content	5.42	%
Volatile matter	73.17	%
Ash Continent	11.71	%
Fixed carbon Continent	9.7	%

Similarly, the Ultimate analysis data, sourced from the ECN Phyllis biomass described in Table 3. The high volatile matter content indicates that sugarcane bagasse ignites readily, facilitating stable furnace once adequately dried. The relatively low nitrogen and Sulphur levels mean that NOₓ and SO₂ emissions are inherently limited compared to coal or heavy fuel oil, aligning with environmental goals. Table 4**.** Shows summary of ultimate analysis results of sugarcane bagasse used as waste biomass fuel in the Metehara Sugar Factory.

**Table 4:** Summary of ultimate analysis result of waste biomass fuel (sugarcane bagasse).

Chemical elements	Mass fraction (wt.%)
Carbon	42.99
Hydrogen	4.97
Oxygen	34.64
Nitrogen	0.22
Sulphur	0.05
Ash content	11.71
Moisture content	5.42
**Total**	**100**

**Fig. 3:**
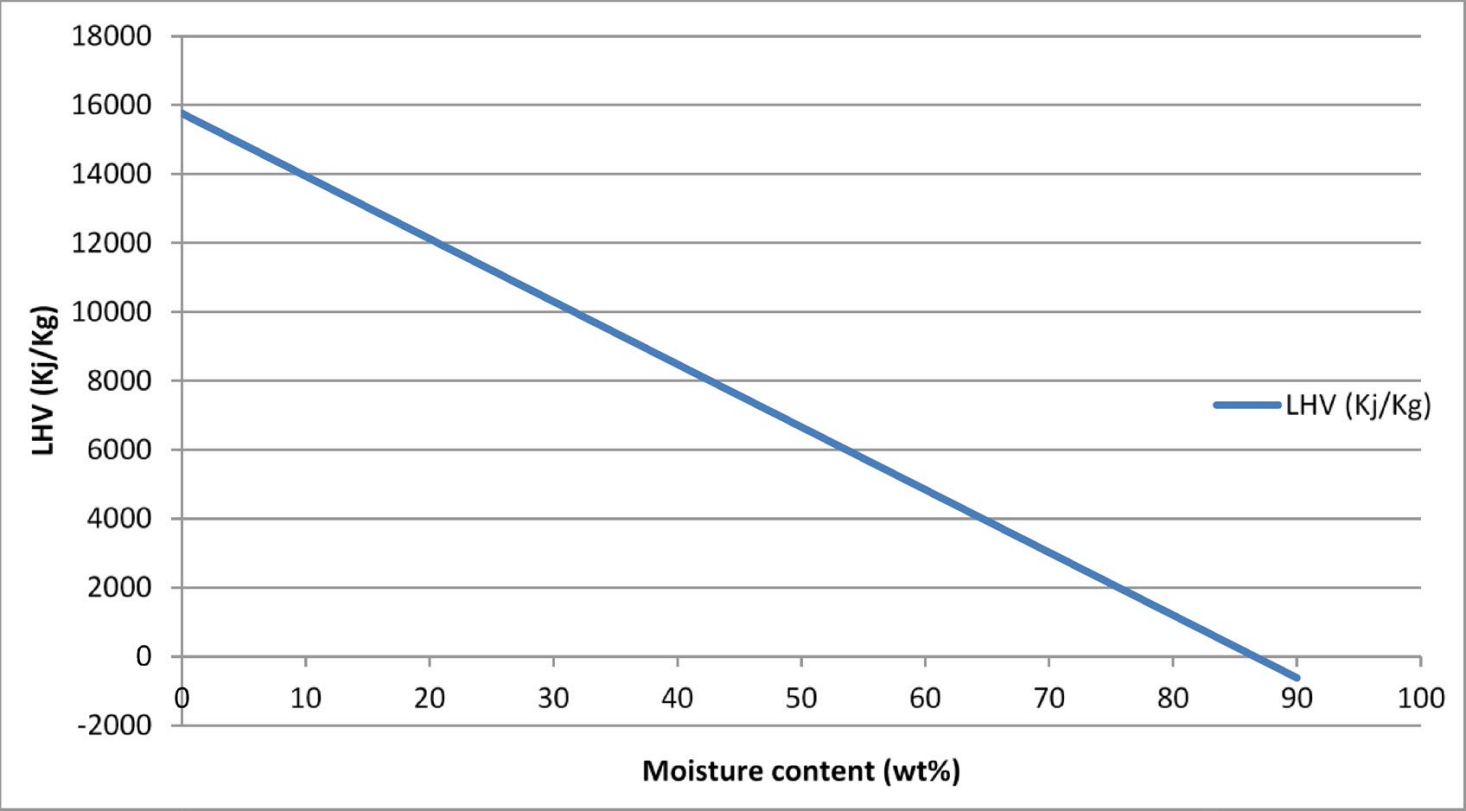
Lower heating value versus moisture content.

A key challenge lies in sugarcane bagasse’s as-received moisture content, typically ~ 46% during factory operations. At this moisture level, the Lower Heating Value (LHV) drops to 7,390 kJ/kg, almost halving the usable energy compared to its dry state (15,760 kJ/kg). Figure 3, clearly shows the steep decline in LHV with increasing moisture, becoming negative above 86.6% moisture content, at which point net energy gain from furnace is impossible.

In essence, this analysis underscores why moisture reduction is central to performance improvement. Drying not only raises heating value and flame temperature but also reduces auxiliary fuel requirements, improves furnace stability, and minimizes boiler fouling, all of which contribute to higher plant efficiency.

### Furnace characteristics

**Fig. 4:**
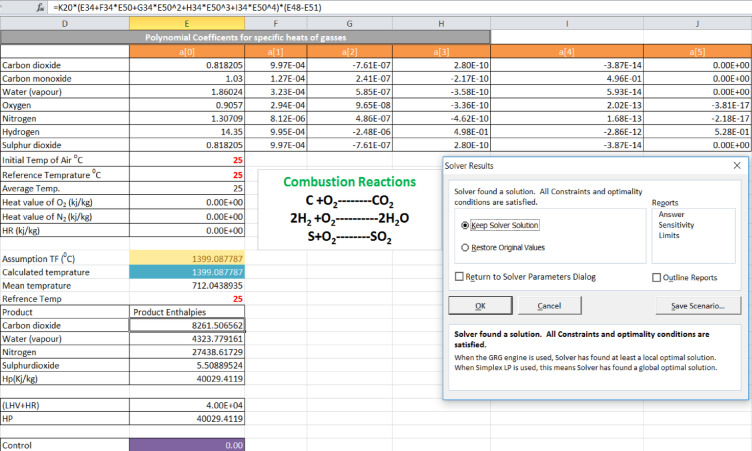
Flame temperature result of a combustion-based biomass steam power plant.

For sugarcane bagasse, the adiabatic flame temperature (T_f_) was designed using an energy balance under steady-state, adiabatic conditions across the boiler furnace. Thus, the T_f_ was calculated using a solver parameter, MS Excel solver, by integrating the energy balance equation (HR + LHV – HP = 0) to zero as described below in Fig. 4.

From the above result, the flame temperature (T_f_) for the combustion-based biomass steam power was 1399.08 °C at a moisture content of 46% (base case).

**Fig. 5:**
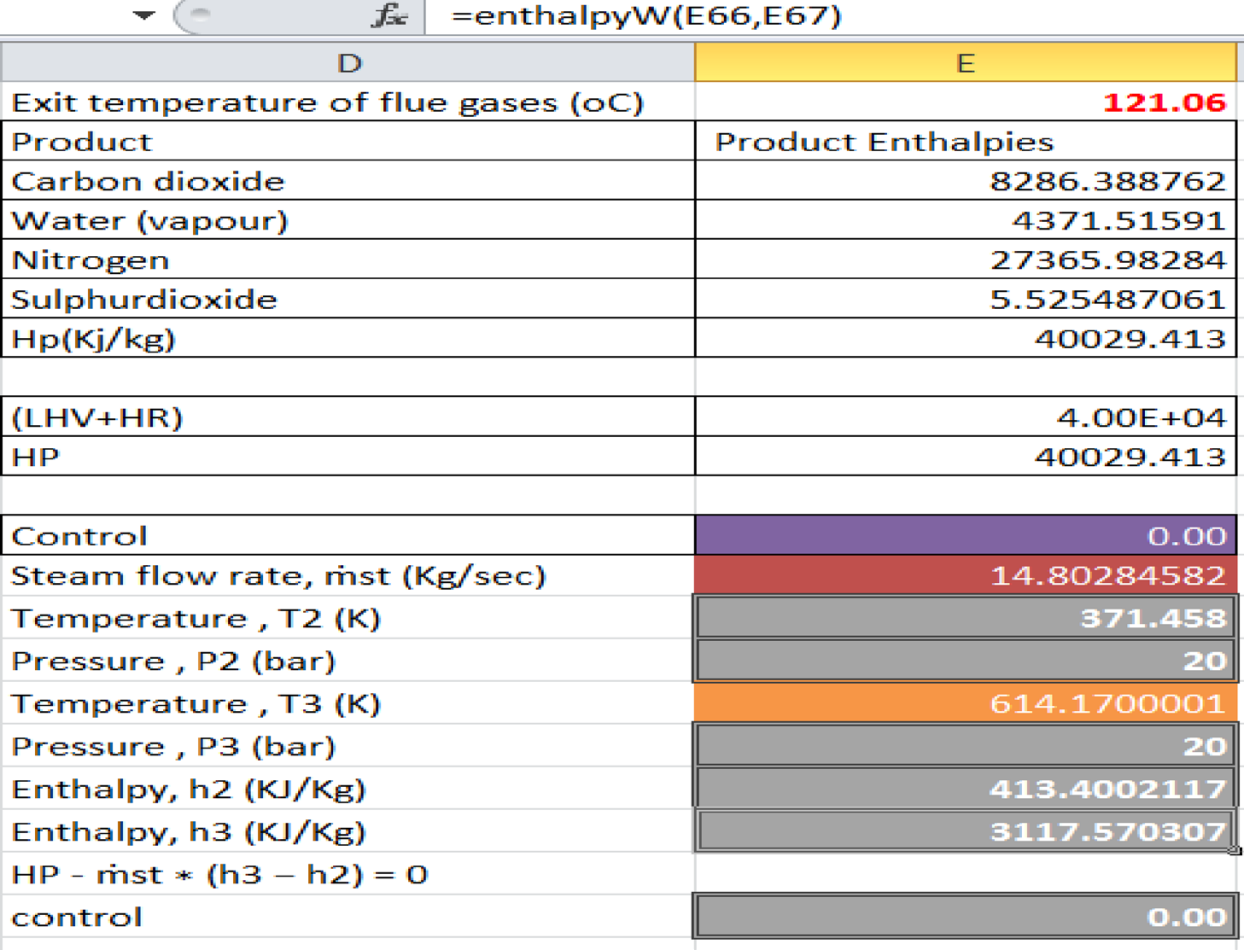
Optimum steam temperature and steam flow rate result from a combustion-based steam power plant.

Similarly, the mass flow rate of the steam (ṁ_st_) and the optimum steam temperature (T_3_) of the combustion-based biomass-fired steam power plant were determined as shown in Fig. 5.

According to the above Fig. 5, the optimum steam temperature (T_3_) and mass flow rate of steam (ṁ_st_) for the combustion-based biomass steam power plant are valued at T_3_ = 341.02 °C and ṁ_st_ = 14.80 kg/se respectively, at a moisture content of 46% (base case).

**Fig. 6:**
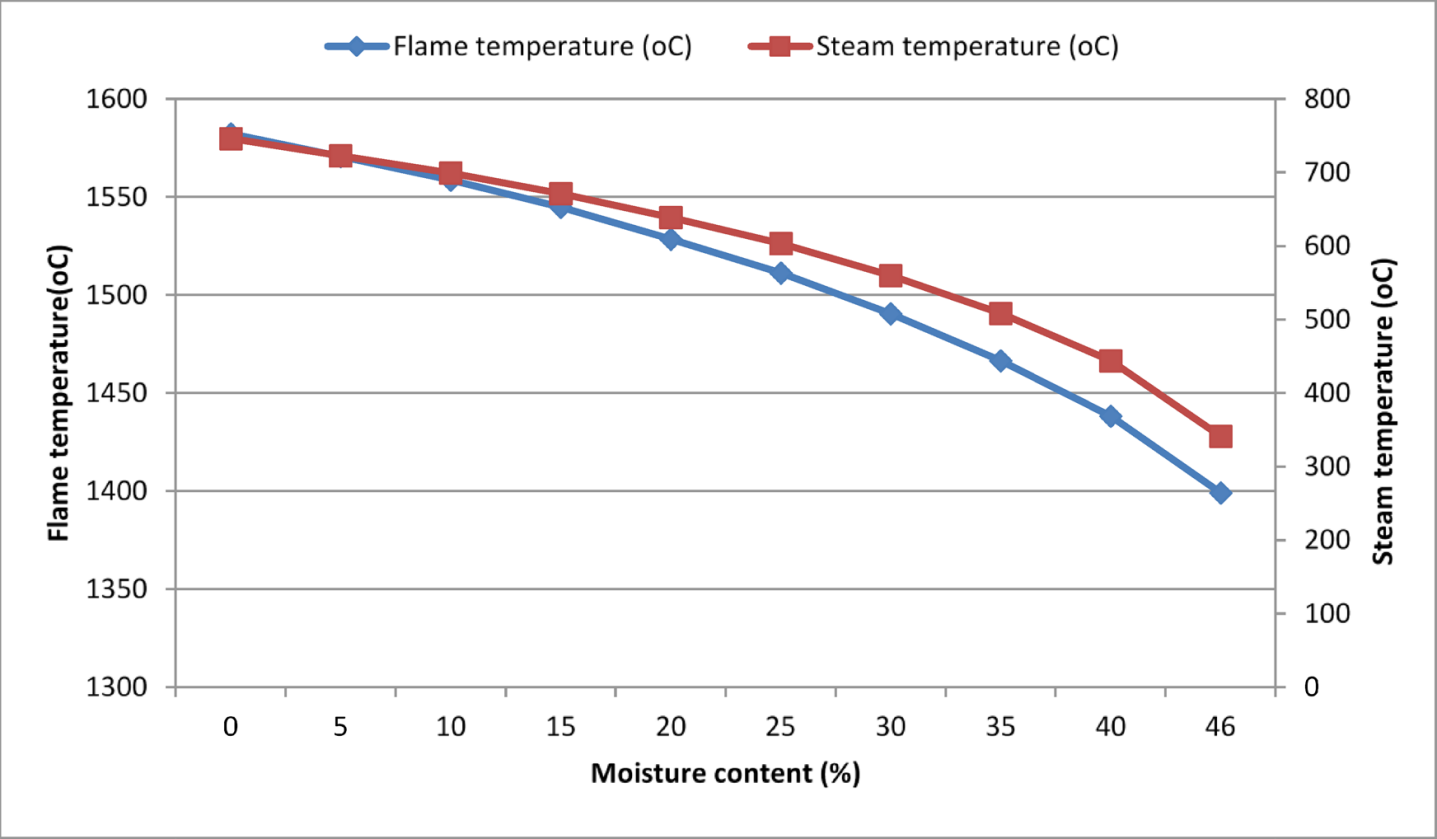
Flame temperature vs. steam temperature vs. moisture content.

The furnace flame temperature of a biomass-fired steam power plant varies as the moisture content of the biomass fuel (sugarcane bagasse) varies, which in turn varies the optimum steam temperature and steam flow rate of a power plant. A numerical analysis was conducted, and graphs were drawn to examine how moisture content affects the furnace process across a range of moisture levels (0–46%), as illustrated in Fig. 6.

The graph above indicates that the biomass waste (sugarcane bagasse) at its lower moisture content exhibits higher furnace temperatures (flame temperature) during combustion, resulting in elevated steam temperatures. This is because biomass waste with low moisture content possesses higher heating values and is combusted more readily. Consequently, to enhance both the power output and thermal efficiency of a biomass-fired steam power plant, it’s necessary to dry or preheat the waste biomass fuel (sugarcane bagasse) before introducing it into the boiler furnace, utilizing various heat integration methods (Table 5).

**Table 5:** Summary result from vapor streams of the Simple Rankine cycle without drying.

States	T(K)	P (bar)	v(m^3^/kg)	h(KJ/kg)	S (KJ/kg. k)	X
1	371.33	0.95	0.001042	411.42	1.28639	0
2	371.55	20	0.001041	413.40	1.28639	–
3	614.17	20	0.136286	3117.57	6.92580	–
4	371.33	0.95	0.096361	2505.50	6.92580	0.92606

### Power plant performance without drying

In this scenario, it’s presumed that steam is introduced into the turbine at 2 MPa and 341.02 °C and condensed in the condenser at a pressure of 95 kPa. Based on the pressure and temperature data provided for the steam power plant, the thermal properties of water under a simple Rankine cycle (base case) are valued as follows:

The calculated temperature, pressure, specific volume, enthalpy, entropy, and quality values for each vapor state are summarized in Table 6. As shown in Table 6, the steam at turbine inlet (State 3) exhibits a high specific enthalpy of 3117.57 kJ/kg, indicating substantial energy availability for power generation. At the turbine exit (State 4), the steam quality is 0.926, confirming the presence of wet steam conditions, which can adversely affect turbine efficiency and blade longevity. These results provide a baseline for evaluating the performance improvements achievable through bagasse drying and enhanced boiler efficiency in subsequent analyses.

**Table 6:** Performance analysis result of the simple Rankine cycle without drying process.

Performed analysis parameters	Results
Wpump, (KJ/kg)	1.985
Wturbine, (KJ/kg)	612.07
Qin, (KJ /kg)	2704.17
Qout, (KJ/kg)	2094.08
Wnet, (KJ/kg)	610.09
Mass flow rate of steam (kg/sec)	14.80
Power output (KW)	9031.01
Thermal efficiency (%)	22.56

The energy and thermal efficiency analysis of a biomass steam power plant relies on the steady-flow energy principle. Hence, the projected analysis results of energy, thermal efficiency, steam mass flow rate, and power generation capacity of the combustion-driven biomass power plant, operating on the simple Rankine cycle (Base case), are tabulated here in Table 6.

**Fig. 7:**
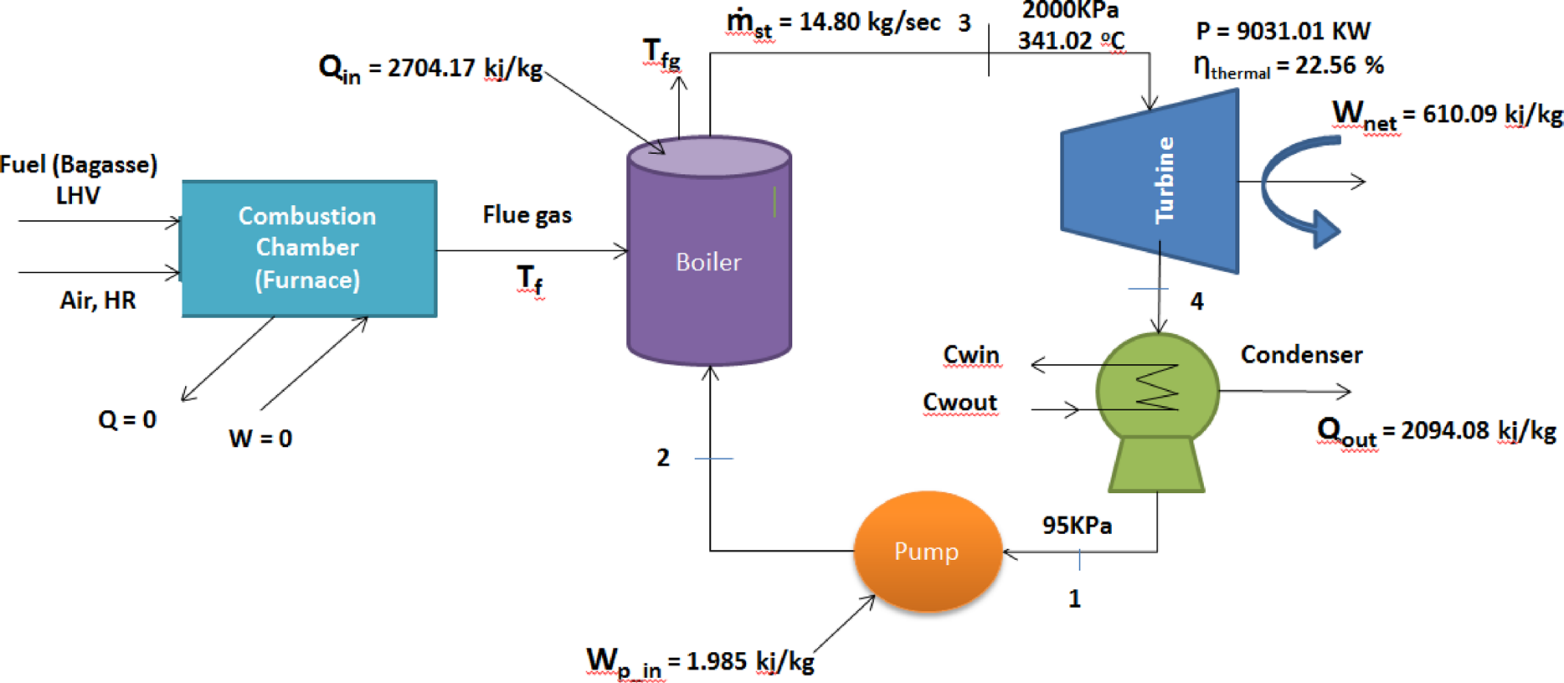
Performance of the simple Rankine cycle without drying.

The schematic diagram, which reveals energy and thermal efficiency analysis result of the combustion-based biomass power plant operating on the simple Rankine cycle (Base case), is also developed as shown in Fig. 7.

### Performance improvement with flue gas-assisted drying

When a drying process was added to the simple Rankine cycle, the flue-gas temperature was determined to be 121.06 °C. The additions to the drying process to the simple Rankine cycle are summarized in Table 7.

**Table 7:** Summary results from vapor streams of the Simple Rankine cycle with the drying process.

States	T(K)	P (bar)	v(m^3^/kg)	h(KJ/kg)	S (KJ/kg. k)	X
1	371.33	0.95	0.001042	411.42	1.28639	0
2	371.55	20	0.001041	413.40	1.28639	-
3	876.34	20	0.200372	3697.88	7.71240	-
4	371.33	0.95	0.102193	2797.59	7.71240	0.98212

The thermal efficiency, steam mass flow rate, and power generation capacity of the combustion-driven biomass power plant, which operates with an added drying process to the simple Rankine cycle (Base case), are shown in Table 8.

**Table 8:** Performance analysis result of the simple Rankine cycle with the drying process.

Performed analysis parameters	Results
Wpump, (KJ/kg)	1.985
Wturbine, (KJ/kg)	900.30
Qin, (KJ/kg)	3284.48
Qout, (KJ/kg)	2386.17
Wnet, (KJ/kg)	898.31
Mass flow rate of steam (kg/sec)	18.49
Power output (KW)	16,609.78
Thermal efficiency (%)	27.35

**Fig. 8:**
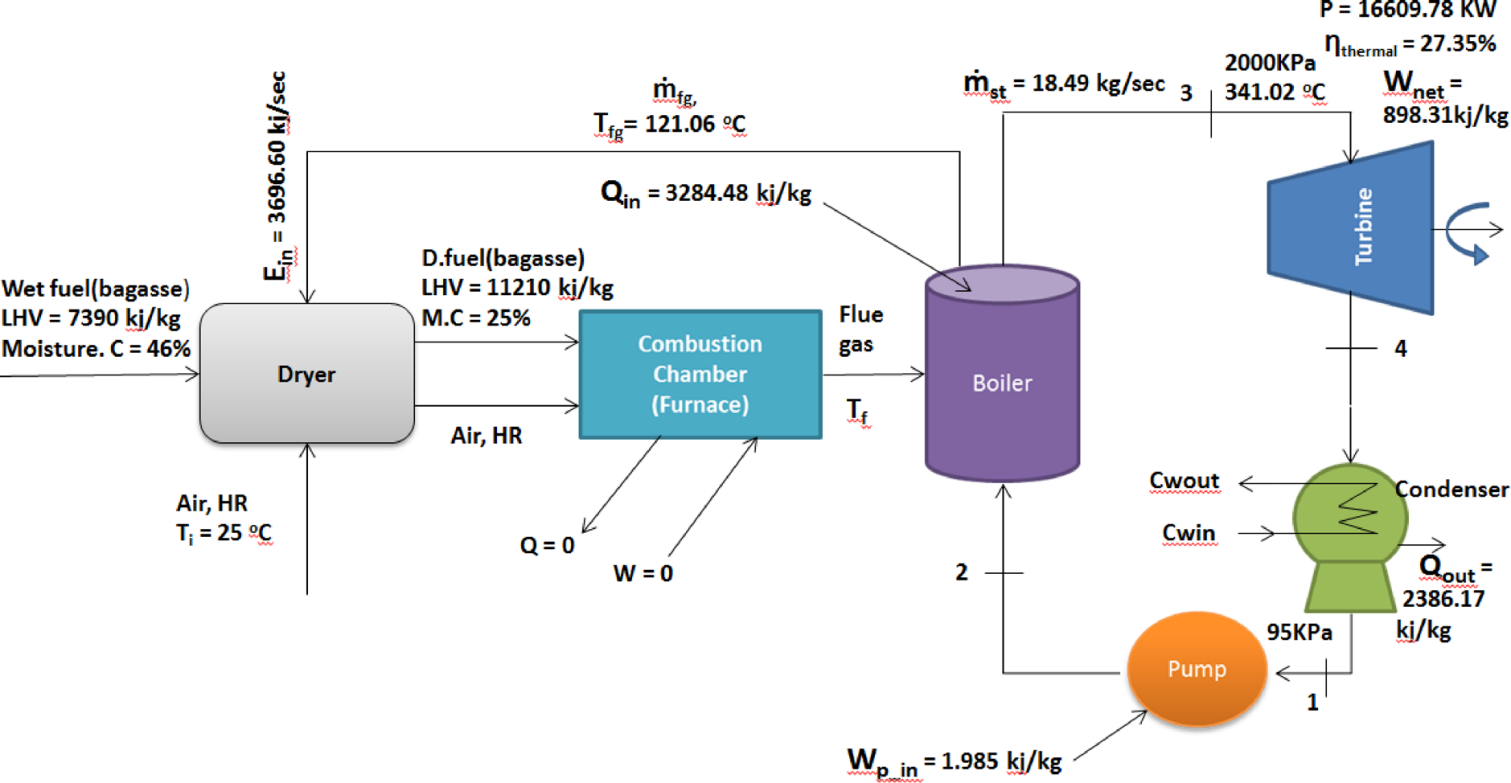
Performance of a simple Rankine cycle with a drying process.

The schematic diagram, which reveals energy and thermal efficiency analysis result of the combustion-based biomass power plant that operates with adding a drying process to the simple Rankine cycle (Base case), is shown in Fig. 8 below.

After Numerical analysis was done, and graphs were drawn to observe how the moisture content affects the combustion-based biomass-fired steam power plant with a range of moisture content variation (0 – 46%).

**Fig. 9:**
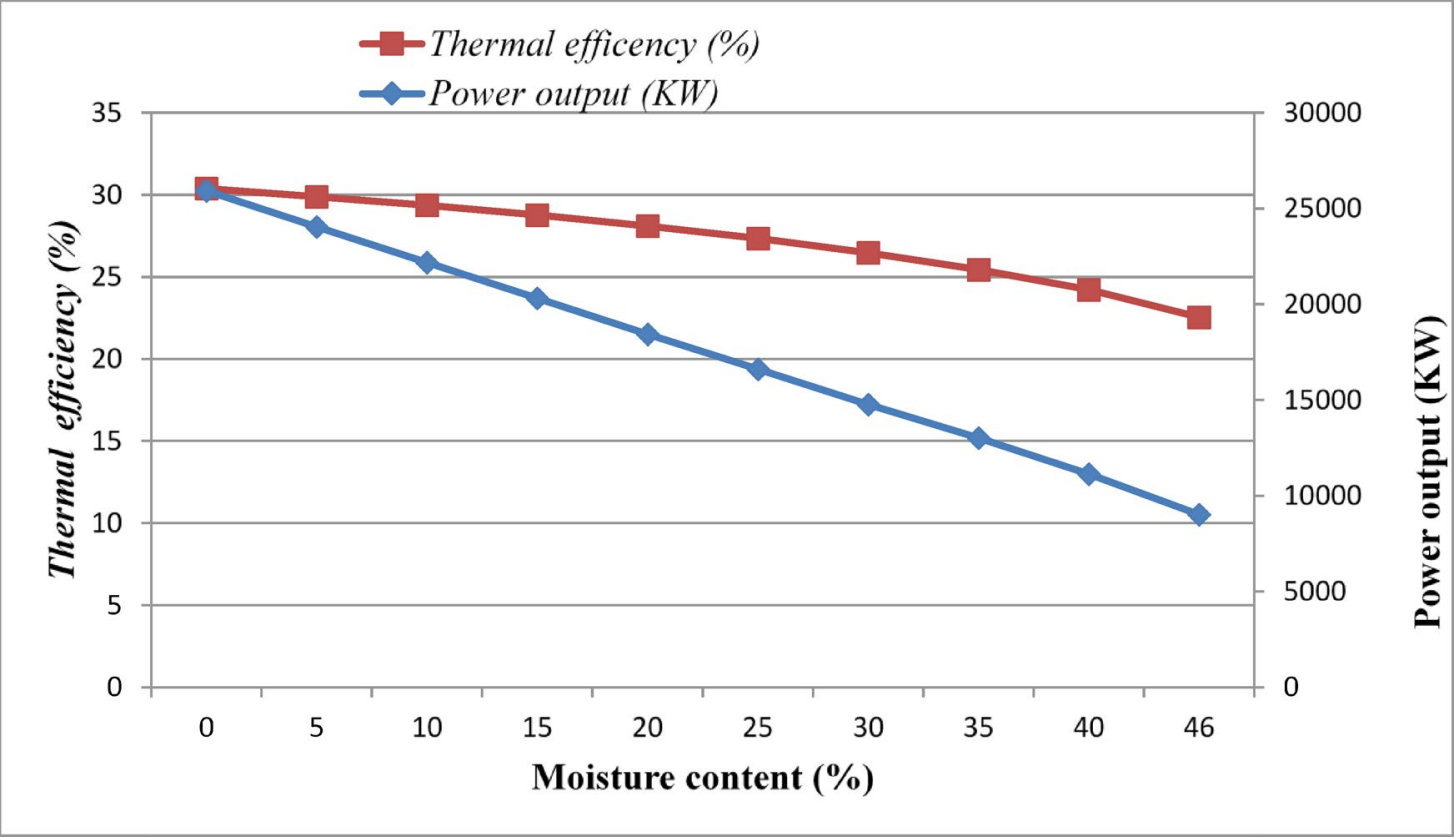
Thermal efficiency vs. power output vs. moisture content.

Figure 9, represents the Thermal efficiency versus power output versus moisture content.

Due to the addition of a drying process to the combustion-based biomass power plant, which operates on the simple Rankine cycle (Base Case), the power generation capacity and the thermal efficiency of the power plant were significantly improved from 9031.01 to 16,609.78 KW and from 22.56 to 27.35%, respectively. Similarly, the amount of mass flow rate of steam was also increased from 14.80 kg/s. into 18.49 kg/s. This is because the drying process removes the excess moisture content of the biomass fuel to increase its lower heating value as it enters the boiler furnace. This increase in the lower heating value of biomass fuel increases the flame temperature, which in turn increases the temperature of steam entering the turbine thereby more power will be harnessed by the turbine blades. As a result, the power output and thermal efficiency of the steam power plant will also be enhanced. Apart from this, drying of biomass fuel also reduces air pollution, flue gas heat loss, operational problems of the boiler, and auxiliary fuels used in the furnace to attain the required power output. The key findings of this paper are highlighted as follows:

- Thermodynamic assessment of a bagasse-fired steam power plant using a simple Rankine cycle
- Flue gas waste heat utilized to reduce biomass moisture prior to combustion
- Net power output increased from 9.03 to 16.61 MW without additional fuel input
- Thermal efficiency improved from 22.56 to 27.35% through fuel moisture reduction
- Results demonstrate a practical efficiency enhancement pathway for sugar industries

### Technical and environmental implications

The integration of flue gas-assisted drying into the sugarcane bagasse-fired steam power plant demonstrates significant technical and environmental advantages, supported by quantitative performance improvements obtained in this study. From a technical perspective, the reduction of fuel moisture content enhances combustion efficiency and overall cycle performance. Specifically, the net power output increased substantially from 9.03 to 16.61 MW, representing an improvement of approximately 83.9%, without additional fuel input. Similarly, the thermal efficiency of the system improved from 22.56 to 27.35%, corresponding to a relative increase of about 21.2%. These improvements are primarily attributed to the increase in the lower heating value (LHV) of the pre-dried bagasse and the reduction in energy losses associated with moisture evaporation during combustion.

From an environmental standpoint, the enhanced efficiency directly contributes to reduced specific emissions per unit of electricity generated. With higher efficiency, less fuel is required to produce the same amount of energy, thereby lowering the overall carbon intensity of the system. Additionally, the recovery and utilization of waste heat from flue gases reduces thermal losses, improving energy utilization within the system. The increase in net power output without additional biomass consumption also implies a reduction in resource demand and associated environmental pressures.

Furthermore, improved combustion conditions associated with lower moisture content lead to more stable flame temperatures and more complete combustion, which are typically associated with reduced emissions of unburned hydrocarbons and particulate matter. Although detailed emission quantification was beyond the scope of this study, the observed efficiency gains and waste heat recovery clearly indicate a more environmentally sustainable operation.

### Techno-economic implications

The integration of flue gas-assisted drying into the sugarcane bagasse-fired steam power plant presents significant techno-economic advantages, particularly when evaluated in the context of existing plant infrastructure and resource utilization. The proposed system utilizes waste heat from boiler exhaust gases, which would otherwise be lost to the environment, thereby minimizing the need for additional external energy input. This makes the drying process inherently energy-efficient and cost-effective compared to conventional drying methods that require dedicated heat sources.

From a technical standpoint, the increase in lower heating value (LHV) due to moisture reduction enhances furnace efficiency, resulting in higher flame temperatures and improved steam conditions. As demonstrated in this study, these improvements lead to a substantial increase in net power output from 9031.01 kW to 16,609.78 kW and an increase in thermal efficiency from 22.56 to 27.35%. Such performance gains imply that more electricity can be generated from the same quantity of biomass fuel, effectively improving the energy conversion efficiency of the system without increasing fuel consumption.

Economically, this enhanced efficiency can translate into reduced operational costs, as less auxiliary fuel is required to maintain desired furnace conditions. In addition, improved boiler performance and reduced moisture-related issues such as fouling and corrosion can lower maintenance costs and extend equipment lifespan. The ability of the plant to generate surplus electricity beyond its internal demand also creates an opportunity for revenue generation through power export to the national grid, thereby improving the financial sustainability of the facility.

Furthermore, since the proposed modification relies on existing waste heat streams and does not require major changes to the core power generation cycle, the capital investment is expected to be relatively low compared to other efficiency enhancement technologies. This makes the approach particularly suitable for implementation in resource-constrained settings, such as agro-industrial facilities in developing countries.

However, it is important to note that a detailed economic evaluation, including capital cost estimation, payback period, and cost–benefit analysis, was not conducted in this study. As highlighted in the limitations section, future work should incorporate comprehensive economic modeling to quantify the financial viability of the proposed system under real operating conditions.

### Implications for National Net-Zero Commitments

The results of this study support national efforts toward net-zero emissions by improving the efficiency of biomass-based power generation. Sugarcane bagasse is a renewable and near carbon–neutral fuel, and its efficient utilization reduces dependence on fossil fuel-based electricity.

The integration of flue gas-assisted drying increases the lower heating value and combustion efficiency, leading to higher power output (from 9.03 to 16.61 MW) without additional fuel input. This reduces emissions intensity per unit of electricity generated and improves overall energy efficiency. Additionally, the potential for surplus electricity generation supports renewable energy expansion and energy access while maintaining a low carbon footprint.

### Limitations of the results

While the results clearly show the benefits of flue gas-assisted drying, several limitations must be acknowledged. Those are modelling assumptions, economic feasibility, environmental quantification, experimental scale, and seasonal and supply variability.

- Modelling Assumptions—The Rankine cycle model was steady-state and did not consider transient effects, equipment degradation, or operational variability.
- Economic Feasibility – The study did not quantify capital and operational costs of installing a drying system, nor did it perform a cost–benefit analysis to determine payback period.
- Environmental Quantification – While reductions in emissions are expected, no detailed life cycle assessment (LCA) or emissions monitoring was conducted.
- *Experimental scale*—Fuel characterization tests were limited and based on single samples; large-scale drying trials were not performed.
- The air–fuel ratio and fuel feed rate were not varied parametrically in this study, and comprehensive sensitivity analysis**,** incorporating variable air–fuel ratios (excess air conditions), fuel feed **rate** variations**,** and Multi-parameter optimization are remaining work for future work.
- Seasonal and supply variability–sugarcane bagasse availability may fluctuate due to agricultural yield variations or plant downtime, which were not modelled in the performance assessment.

Addressing these gaps is essential to advance the present work from theoretical modelling toward full-scale experimental implementation. Future research should integrate dynamic operational modelling with comprehensive experimental validation, including pilot- or field-scale trials, to assess system performance under real-world conditions. In addition, detailed cost–benefit analysis and environmental impact evaluation will be necessary to support large-scale deployment, ensure economic viability, and provide a long-term perspective on the sustainability of the proposed approach.

## Conclusions

This study evaluated the impact of flue gas-assisted drying on the performance of a sugarcane bagasse-fired steam power plant at Metehara Sugar Factory using a steady-state thermodynamic modelling approach. The results indicate that the available sugarcane bagasse resource (approximately 170,820 tons/year) is adequate to support continuous small-scale power generation, although its effectiveness is strongly influenced by fuel moisture content.

The analysis shows that high as-received moisture content (~ 46%) reduces the lower heating value of bagasse and limits combustion temperature, which in turn constrains steam conditions and overall cycle performance. Incorporating flue gas-assisted drying, modelled as a waste heat recovery process, improved fuel quality and increased the estimated turbine inlet temperature. Under the modelled conditions, net power output increased from 9.03 to 16.61 MW, while thermal efficiency improved from 22.56 to 27.35%. These results suggest that moisture reduction can significantly influence system performance; however, they are based on idealized assumptions and should be interpreted accordingly. In addition to performance improvements, the use of waste heat for drying has the potential to enhance energy utilization within the plant. Improvements in combustion conditions may contribute to more stable operation and reduced relative heat losses, although detailed emission reductions and operational impacts were not quantitatively assessed in this study.

Despite these promising findings, several limitations should be emphasized. The analysis is based on steady-state modelling and does not account for transient operation, system losses, or equipment degradation. Economic feasibility, including capital investment and payback period, was not evaluated, and environmental benefits were inferred rather than quantified through detailed emission or life-cycle assessment. Furthermore, the results have not been validated through pilot-scale or full-scale experimental implementation.

Therefore, while flue gas-assisted drying shows potential as an efficiency improvement strategy for biomass-fired power systems, further work is required to validate these findings under real operating conditions. Future studies should incorporate dynamic modelling, experimental verification, and comprehensive techno-economic and environmental assessments to better establish the practical applicability of the proposed approach.

## Supplementary Information

Below is the link to the electronic supplementary material.

Supplementary Material 1

Supplementary Material 2

## Data Availability

Data Availability Statement: The datasets generated and analyzed during the current study are included in this published article and its supplementary information files. Additional supporting data related to plant operating parameters and fuel characterization were obtained from Metehara Sugar Factory and are available from the corresponding author upon reasonable request, subject to institutional permission. Thermodynamic modelling data were generated using Engineering Equation Solver (EES) and Microsoft Excel, and all governing equations and input parameters are provided in the Materials and Methods section to ensure reproducibility.

## Abbreviations

*AFT *: Adiabatic flame temperature
*A/F *: Air to fuel ratio
*A *: Ash content
*BFB*: Bubbling fluidized bed
*CFB*: Circulating fluidized bed
*CHP*: Combined heat and power
*Cp*: Specific heat capacity
*E*_*in*_: Energy entering the system
*E*_*out*_: Energy leaving the system
*ECN*: Energy center of Netherlands
*EES*: Engineering equation solver
*ESC*: Ethiopia sugar corporation
*FC*: Fixed carbon
*GOE*: Government of Ethiopia
*GCV*: Gross calorific value
*h *: Enthalpy
*HHV*: Higher heating value
*HAD*: Hot air dryer
*HR*: The heat of reactants
*HP*: The heat of combustion products
*IAPWS-IF*: International association for properties of water and steam-industrial formulation
*K*: Kelvin
*KPa*: Kilo pascal
*LHvap*_*w*_: Latent heat of vaporization of water
*LHV*: Lower heating value
*m*_*1*_: Intial weight of sample biomass fuel
*m*_*fg*_: Mass of flue gas
*m*_*w*_: Mass of water removed
$\dot{m}$_*fuel*_: Mass flow rate of biomass fuel
$\dot{m}$_*cw*_: Mass flow rate of cooling water
$\dot{m}$_*in*_: Mass entering the system
$\dot{m}$_*out*_: Mass leaving the system
*MPa*: Mega pascal
*MW *: Mega watt
*Mss*: Millable sugar cane stalks
*M*: Moisture content
*MSW*: Municipal solid waste
*m*_*2*_: Weight of sample biomass fuel left/loss
*Nm *: The mole fraction of mixture
*Ni *: The mole fraction of species
*NCV*: Net calorific value
*Pm*: The partial pressure of mixture
*Pi*: The partial pressure of species
*P*: Power out put
*Q*_*in*_: The heat supplied to the system
*Rtot*: Total amount of residue produced
*Rep*: Residue available for energy production
*Rop*: Residue used for other purposes
*RPR *: Residue to product ratio
*s*: Entropy
*STP*: Standard temperature and pressure
*SSD*: Super—heated steam dryer
*Tadp*: Acid dew point temperature
*T*_*fg*_: Exit temperature of flue gas
*T*_*f*_: Flame temperature
*T*_*i*_: Initial temperature
*T*_*cw*__*,in*_: Inlet temperature of cooling water
*T*: Mean temperature
*T*_*cw*__*,out*_: Outlet temperature of cooling water
*T*_*3*_: Optimum steam temperature
*Tsat*: Saturation temperature
*v*: Specific volume
*VOC*: Volatile carbon
*VM*: Volatile matter
*W*: Work done by the system
*W*_*p_in*_: Work done by the pump
*W*_*t_out*_: Work done by the turbine
*W*_*net*_: Network done
*X*: Vapor fraction/quality of steam
*η*_*thermal*_: Thermal efficiency
