# Supplementary material for: Performance enhancement of a sugarcane bagasse-fired steam power plant through flue gas-assisted drying: a case study of Metehara Sugar Factory, Ethiopia
Source: Sci Rep. 2026 Apr 25;16:17160. doi: 10.1038/s41598-026-50724-7 (PMC13234332; doi:10.1038/s41598-026-50724-7)
Supplement: Supplementary file 1 — Supplementary Material 1 [file 41598_2026_50724_MOESM1_ESM.docx]

***Performance Enhancement of a Sugarcane Bagasse-Fired Steam Power Plant through Flue Gas-Assisted Drying: A Case Study of Metehara Sugar Factory, Ethiopia***

***APPENDIXES***

***Appendix 1: Molecular weight of chemical elements, combustion products, and air composition***

| ***Chemical elements (reactants)& combustion products*** | ***Molecular weight (Kg/kmol)*** | ***Air composition (wt.%)*** |
| --- | --- | --- |
| *Oxygen(O_2_)* | *32* | *21* |
| *Nitrogen(N_2_)* | *28* | *79* |
| *Carbon (C)* | *12* | *-* |
| *Hydrogen(H_2_)* | *2* | *-* |
| *Sulphur* | *32* | *-* |
| *Water (H2O)* | *18* | *-* |
| *Carbon dioxide (CO2)* | *44* | *-* |
| *Sulphur dioxide (SO2)* | *64* | *-* |

***Appendix 2: Oxygen requirement of biomass fuel (bagasse)***

| ***Elemental composition*** | ***Composition***  ***(wt. (%)*** | ***Mass fraction***  ***(Kg/sec)*** | ***Oxygen requirement (kg/sec)*** |
| --- | --- | --- | --- |
| *Carbon* | *24.54* | *1.3292* | *3.5447* |
| *Hydrogen* | *2.84* | *0.1538* | *1.2307* |
| *Sulphur* | *0.03* | *0.0016* | *0.0016* |
| *Nitrogen* | *0.12* | *0.0065* | *-* |
| *Oxygen* | *19.78* | *1.0714* | *-1.0714* |
| *Ash* | *6.69* | *0.3624* | *-* |
| *Moisture content* | *46* | *2.4917* | *-* |
| ***Total*** | ***100*** | ***5.4167*** | ***3.7056*** |

***Appendix 3: Flame temperature of biomass fuel (bagasse) at different moisture content***

| ***Moisture content (%)*** | ***C***  ***(%)*** | ***H***  ***(%)*** | ***O***  ***(%)*** | ***N***  ***(%)*** | ***S***  ***(%)*** | ***Ash (%)*** | ***Combustion chamber flame temperature (^o^C)*** |
| --- | --- | --- | --- | --- | --- | --- | --- |
| *46* | *24.54* | *2.84* | *19.78* | *0.12* | *0.03* | *6.69* | *1399.08* |
| *40* | *27.27* | *3.16* | *21.97* | *0.14* | *0.03* | *7.43* | *1438.21* |
| *35* | *29.54* | *3.42* | *23.81* | *0.15* | *0.03* | *8.05* | *1466.59* |
| *30* | *31.82* | *3.68* | *25.64* | *0.16* | *0.03* | *8.67* | *1490.42* |
| *25* | *34.09* | *3.94* | *27.47* | *0.17* | *0.04* | *9.29* | *1511.24* |
| *20* | *36.36* | *4.21* | *29.31* | *0.18* | *0.04* | *9.90* | *1528.57* |
| *15* | *38.63* | *4.47* | *31.14* | *0.2* | *0.04* | *10.52* | *1544.74* |
| *10* | *40.91* | *4.73* | *32.96* | *0.21* | *0.05* | *11.14* | *1558.63* |
| *5* | *43.18* | *5* | *34.79* | *0.22* | *0.05* | *11.76* | *1570.58* |
| *0* | *45.45* | *5.26* | *36.63* | *0.23* | *0.05* | *12.38* | *1582.28* |

***Appendix 4: Optimum steam temperature and steam flow rate of biomass fuel (bagasse) at different moisture content***

| ***Moisture content (%)*** | ***Mass flow rate of steam, ṁ_st_ (kg/sec)*** | ***Steam temperature, T_3_ (^o^C)*** |
| --- | --- | --- |
| *46* | *14.80* | *341.02* |
| *40* | *15.67* | *443.99* |
| *35* | *16.55* | *508.49* |
| *30* | *17.50* | *559.8* |
| *25* | *18.49* | *603.19* |
| *20* | *19.51* | *638.64* |
| *15* | *20.52* | *671.28* |
| *10* | *21.56* | *699.01* |
| *5* | *22.61* | *722.93* |
| *0* | *23.63* | *746.24* |

***Appendix 5: Power output and thermal efficiency of biomass fuel (bagasse) at different moisture content***

| ***Moisture content (%)*** | ***Power output (KW)*** | ***Thermal efficiency (%)*** |
| --- | --- | --- |
| *46* | *9031.01* | *22.56* |
| *40* | *11130.34* | *24.23* |
| *35* | *13018.43* | *25.44* |
| *30* | *14758.84* | *26.46* |
| *25* | *16609.78* | *27.35* |
| *20* | *18439.64* | *28.09* |
| *15* | *20309.39* | *28.78* |
| *10* | *22179.05* | *29.37* |
| *5* | *24037.08* | *29.88* |
| *0* | *25929.86* | *30.38* |
