# Supplementary material for: Performance enhancement of a sugarcane bagasse-fired steam power plant through flue gas-assisted drying: a case study of Metehara Sugar Factory, Ethiopia
Source: Sci Rep. 2026 Apr 25;16:17160. doi: 10.1038/s41598-026-50724-7 (PMC13234332; doi:10.1038/s41598-026-50724-7)
Supplement: Supplementary file 2 — Supplementary Material 2 [file 41598_2026_50724_MOESM2_ESM.docx]

## *Literature review*

## *Performance evaluation of steam power plant*

*Performance analysis of combustion process utilizing bagasse as fuel was conducted (Amor rat, 2013). The study was to investigate the effect of moisture content on key composition parameters which include gross calorific value (GCV), net calorific value (NCV), water evaporation load, Adiabatic flame temperature (AFT) and equilibrium analysis (exhaust gas compositions). It was found that with increased bagasse moisture content, the bagasse compositions (C, H, O & N) are decreased and results in a decrease in gross calorific value (GCV) and net calorific value (NCV). The adiabatic flame temperature also decreases with increasing moisture content and can cause incomplete combustion and undesirable reaction products as the moisture content is too high because most of the heat from bagasse combustion is used for evaporation. This finding indicated that drying increases heating value, adiabatic flame temperature; reduce pollution and operational problems which consequently results in overall efficiency improvement of the combustion process (12).*

*An energy analysis was conducted on a steam power plant integrated with dryer that works on Rankine cycle, featuring components such as a condenser, steam turbine, dryer, boiler, and feed water pump (Han Song, 2012). Using the constant flow approach, each cycle component operates as a steady flow device. The analysis yielded insights into several key parameters: turbine power output, pump power consumption, energy needed for drying biomass fuel, heat exchange within the cycle, and overall thermal efficiency. It was found that increasing boiler pressure and turbine inlet temperature enhances the plant's energy efficiency. Incorporating drying operations with proper heat integration in to steam power plant significantly improve the cycle's overall energy efficiency (13).*

*Thermodynamic properties evaluation of steam power plant integrated with dryer that works on Rankine Cycle was performed, focusing on key components including the boiler, turbine, feed water pump, condenser, and dryer, all treated as steady flow devices (Ming Liu, 2017). The analysis determined that the plant operates at a thermal efficiency of approximately 23.1% and 24.31% for using steam and flue gas dryer respectively. It was observed that the exergetic efficiency of the heat exchange process in the boiler is equal to that of the conventional biomass power plant. The analysis further indicated that integrating proper drying techniques and optimizing heat exchange can enhance the overall exergetic efficiency by over 1.5% and 3% when steam extraction and flue gas are used as drying heat source respectively, compared to processes that do not incorporate drying (5).*

## *Effect of adding drying process in a steam power plant*

*Steam power plant is a thermal power station in which heat energy is converted in to electric power using steam that spins a steam turbine which in turn drives an electrical generator. Combustion is a key process in steam power plant in which different types of fuels burn in furnace or boiler to generate heat, boil water and produce steam. The big difference in the design of steam power plant results from different heat sources. Nuclear, biomass and to a great extent fossil fuels (coal, natural gas and petroleum) are the sources of heat in combustion (14). However, due to the rapid depletion of fossil fuels and the concern of global warming there is a growing demand and dramatic shift towards renewable energy source. In this regard biomass is the most reliable option and used as alternative to fossil fuel in producing power. Biomass fired steam power plant use renewable fuels such as fire wood, sorghum husk, rice husk, sugarcane bagasse, algae, coconut and other agricultural waste and municipal solid wastes burnt in boiler to generate high pressure steam (15).*

*
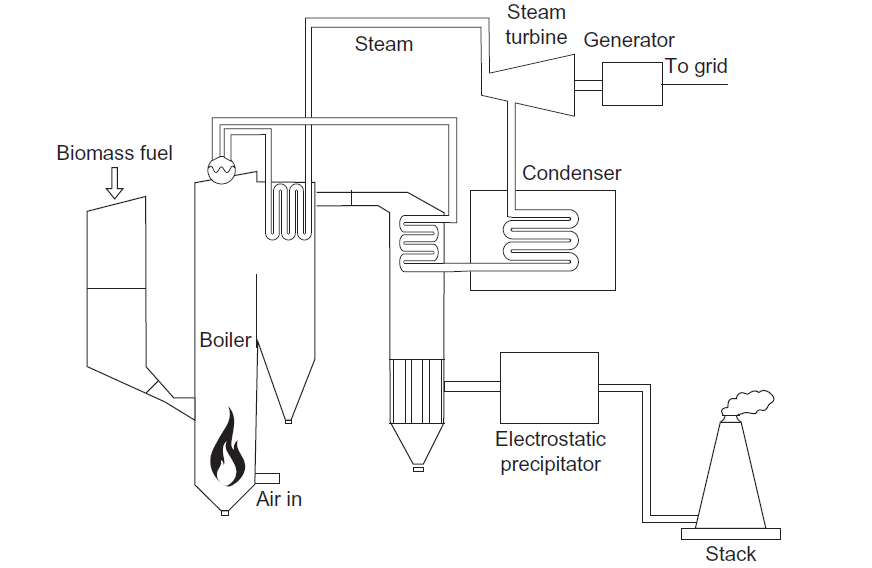
*

*Figure 1. Schematic diagram of biomass fired steam power plant (16)*

*Regardless of the different types of fuel used steam power plant works based on ideal rankine cycle in which power is produced by alternately vaporizing and condensing a working fluid water. As shown in the diagram below rankine cycle has four processes, in which the state of working fuel changed in each process in cycle. In process 1-2_s_ first water is pumped isentropically from low to high pressure by externally powered pump. In process 2_s_-3 the high pressure water goes to boiler and changed to saturated vapor through heating at constant pressure by an outside heat source biomass. The saturated vapor then expands through a turbine isentropicaly in process 3-4_s_ to produce power while reducing the temperature and pressure of the vapor. Finally in process 4_s_-1the vapor goes to a condenser and condensed to become a saturated liquid. The water goes to the pump again and the cycle repeats itself.*

**

*Figure 2. the T-S and schematic diagram of simple Rankine cycle (14)*

*The efficiency of a steam power plant that works on Rankine cycle can be improved either through raising the average temperature at which heat is transferred to the boiler's working fluid or lowering the average temperature at which heat is rejected from the condenser's working fluid. This can be done in three ways; these are lowering the condenser pressure, superheating the steam to high temperatures and increasing the boiler pressure respectively (14).*

*The efficiency of a simple Rankine cycle can also be improved by drying a biomass fuel prior to combustion in a steam power plant. Drying reduces the high moisture content of most raw biomass materials (usually 30-60%) to optimum level (usually 10-15%) and improves properties in terms of energy use. Drying enhances the calorific value of biomass fuels which in turn increase the combustion temperature (flame temperature) and the temperature of steam with reduction in stack loss. This significantly improves the efficiency of the steam power plant. Biomass can be dried in stand-alone dryer or a dryer integrated with steam power plant. Different heat sources can be used to dry biomass such as hot furnace, flue gas, solar radiation, gas turbine exhaust gases, high pressure steam from a steam cycle plant and warm air from air cooled condenser in a steam cycle plant. Dryers integrated with steam power plant are preferable as they use the waste heat produced by steam power plant units as drying mediums for energy saving process (17).*

*
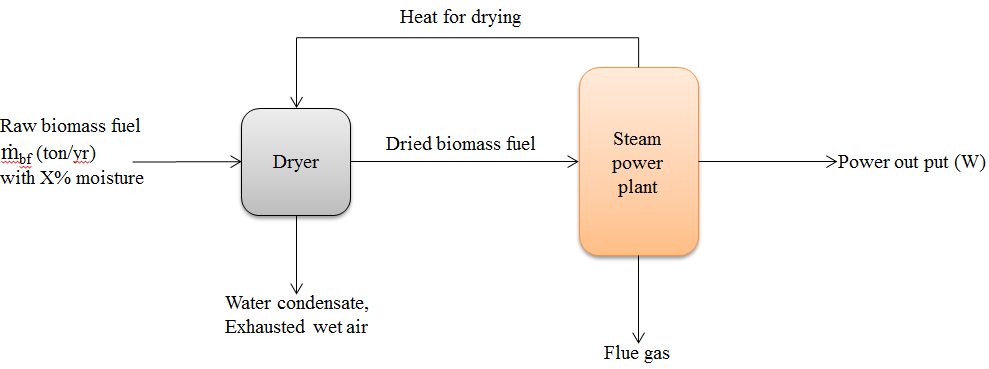
*

*Figure 3. simpliﬁed process ﬂow diagram of steam power plant integrated with dryer*

*Modeling and optimization of drying operation utilizing microalgae for power generation was conducted. The simulation study used hot air dryer (HAD) and a combination of hot air dryer and super-heated steam dryers (SSD) to reduce the moisture content of microalgae using low pressure steam from the power plant to which they are integrated with. The analysis result indicated that drying raises the average temperature at which heat is transmitted to the steam which result in 5-6.5% increase in cycle efficiency and 45-48% decrease in fuel usage for using HAD and a combination of HAD and SSD respectively (18).*

*An analytical study of wood chip drying through the using of a mobile rotary dryer was done. The study indicated that in addition to reduction in the caloriﬁc value of the biomass fuel with a negative eﬀect on the eﬃciency of power generation in combustion process; higher biomass moisture content causes an increase of CO and volatile carbon (VOC) emission as well as the formation of carcinogenic compounds from combustion which may cause severe diseases, air and soil contaminations. The analytical study also showed that some characteristics of the biomass fuel such as initial moisture content, particle size distribution, bulk density inﬂuence the technical parameters of the drying process including airﬂow temperature, air flow rate, and air flow speed, hence, the energy demand for drying. The analysis result obtained suggest that an increase in the eﬃciency of the thermal insulation of the mobile dryer, and heat integration application of the mobile dryer in a small farm, for the recovery of exhaust gases from thermal power plants so as to save the external energy required for drying the biomass fuel (19).*

*Drying biomass offers considerable benefits for combustion such as increased boiler efficiency, lower flue gas emissions and improved boiler operations. Drying is however an energy-intensive pre-treatment. A comparative evaluation of biomass drying process was performed. The study indicated that Large amounts of low-grade waste heat from a range of process industries is widely available, in both their cooling water, at about 90°C, and their flue gases, at 250-400°C which can dry a biomass fuel and fulfill the energy demand of drying process, hence save the cost of energy. The analysis result further ensure that using flue gas as drying heat source would result in lower capital costs than using superheated steam (20).*

## *Biomass to energy conversion methods and technologies*

*Biomass conversion involves transforming organic materials into energy for heat, power or combined heat and power (CHP) generation. Nowadays, there are several technological options available to make use of biomass as a source of renewable energy. Depending on the composition of the fuel, biomass can be converted into energy using methods such as combustion, gasification, pyrolysis, extraction, fermentation, and anaerobic digestion. It can also be transformed into different energy carriers but in general, the process of converting biomass into energy falls into three main categories: thermochemical conversion, biochemical conversion, or mechanical conversion. The key factors that influence the process of converting biomass into useful forms of energy through thermochemical, biochemical, and mechanical conversion methods are the type and amount of biomass feedstock; the desired form of energy, i.e., end-use requirements; environmental regulations; economic feasibility; and project-specific factors. In many situations it is the form in which the energy is required that determines the process route, followed by the available types and quantities of biomass (21).*

*
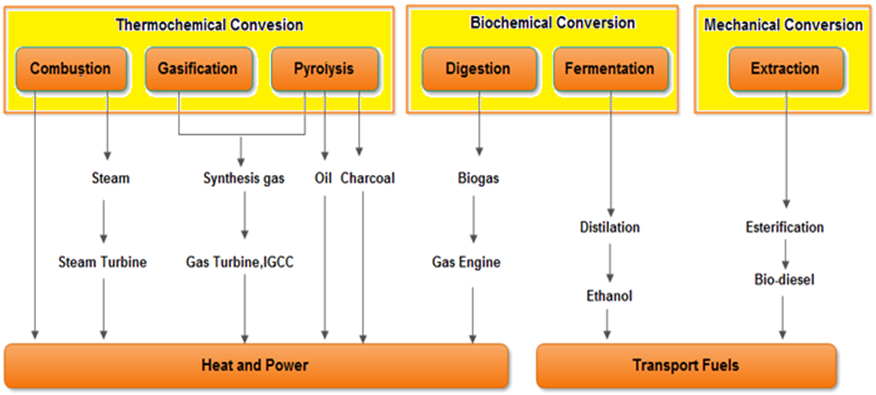
*

*Figure 4. Biomass to Energy conversion methods (22)*

*Thermochemical conversion is one method of harnessing the energy stored in biomass to generate heat, power or combined heat and power (CHP). This conversion method is commercially more attractive due to its improved ﬂexibility, product selectivity, quicker conversion degree, higher process effectiveness, and different market for byproducts. The three fundamental thermochemical processes are combustion, gasification, and pyrolysis. The type and amount of biomass feedstock; the desired form of energy, i.e., end-use requirements; environmental regulations; economic feasibility; and project-specific factors are the key factors for the selection of the conversion process (23).*

*In the combustion process, biomass undergoes immediate burning in the presence of air to convert its stored chemical energy in to heat, mechanical power, or electricity using various items of process equipment (e.g. furnaces, boilers, steam turbines, generators, etc.). Burning biomass is possible across all types, but practically feasible combustion occurs only when the biomass contains less than 50% moisture, unless it has been pre-dried. Apart from converting biomass's stored energy into power, burning waste biomass also decreases its volume and weight, thus lowering the requirements for handling and landfill space. Biomass can also burn (co-fired) together with other fuels which is an emerging attractive conversion route (23).*

*Co-firing refers to the practice of using supplementary fuel alongside a primary fuel in high-efficiency boilers. It is the simplest and economical efficient biomass to energy conversion approach. Recently, co-firing has been applied by using coal as the primary fuel and adding biomass typically at levels ranging from 1% to 20% by weight. Co-firing is implemented when the biomass has high moisture content and is less reliable. Co-firing offer benefits include low capital investment, low environmental emission and high conversion efficiency compared to biomass only power plant (24).*

*Pyrolysis and gasification are the two other thermochemical conversion processes each with their own distinctive feature. Gasification is the transformation of biomass into a flammable gas mixture through the partial burning of biomass at elevated temperatures, usually between 800 and 900 ^o^C which is utilized as a fuel for gas engines and gas turbines and as raw material for manufacturing of chemicals. Whereas: Pyrolysis involves converting biomass into liquid, solid, and gaseous components by heating it to approximately 500^o^Cin the absence of air. It is primarily used to generate a liquid fraction, utilized as fuel in engines and turbines and as a raw material in refineries with an efficiency of up to 80% (23).*

*Another important biomass to energy conversion technology is combined heat and power (CHP) also known as co-generation. Co-generation is the process of generating electricity and heat simultaneously from a single energy source, aiming to achieve greater efficiency compared to generating them separately. It is cost- efficient and crucial solution for decreasing CO_2_ emissions. Any facility requiring both electricity and thermal energy on a continuous basis is well-suited for implementing CHP. However, the feasibility of biomass CHP facilities is generally influenced by the cost and accessibility of biomass feedstock, alongside energy prices (25).*

*Among the biomass to energy conversion routes combustion is the most mature and commonly applied process due to its low cost and high reliability. It is widely available technology implemented across a wide range of scales, from few MW to 3000 MW with 90% share of world’s bioenergy production. However, combustion of biomass has low efficiency compared to that of conventional coal power plant due to relatively high moisture content, low heating value of biomass and flue gas heat loss. The efficiency of biomass power plant can be enhanced either by using dry biomass fuel or by implementing a waste heat recovery system to capture and utilize the energy in the flue gas for drying biomass fuel prior to combustion process (23).*

*Different technologies are available for burning biomass fuels but, the most common ones are stoker and fluidized bed boilers each differ in their biomass particle size, moisture content requirement and power generation capacity. Stoker boilers are typically used more frequently with smaller capacity boilers, lower moisture content, and larger fuel sizes. They can be categorized as either overfed or underfed, with overfed stokers introducing fuel from above and underfed stokers introducing fuel from below. Stoker boilers are advantageous in that they are simple, affordable, consume less fuel and respond quickly to changes in heat demand. On the other hand, they are inconvenient in that their performance is sensitive to moisture content and lack separate fans for primary and secondary combustion air, making it challenging to control combustion temperature and the formation of NOx emissions (26).*

*Fluidized bed boilers operate by suspending fuel particles in a bed of hot sand or similar mineral which is initially heated by an oil or gas burner and kept fluidized by jets of air from below, allowing for efficient combustion of the fuel particles. This ensures constant mixing of the fuel with the main airflow and thus more effective combustion, especially for lower quality and variable moisture content fuels. Secondary air is also introduced above the fluidized bed to guarantee complete combustion. Fluidized bed boilers can be bubbling fluidized bed (BFB) or circulating fluidized bed boilers (CFB).*

*
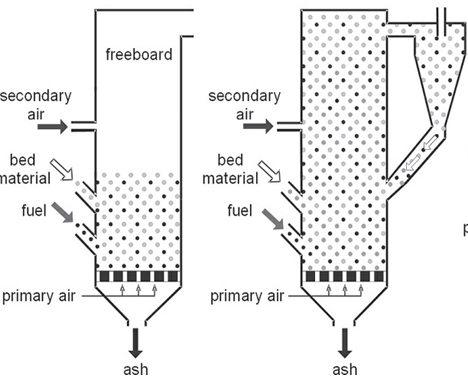
*

*Figure 5. working principle of bubbling fluidized bed and circulating fluidized bed boilers (27)*

*Bubbling fluidized bed (BFB) boilers are typically selected for smaller-scale applications, whereas circulating fluidized bed (CFB) boilers are commonly employed in larger-scale operations. In general fluidized bed boilers are good in reducing SO_2_ and NO_x_ emissions from coal burning as they are designed more specifically to burn biomass fuels (26).*

**References**

1. Paulina Drożyner, Wojciech Rejmer, Piotr Starowicz, Andrzej Klasa, Krystyna A. Skibniewska**.** Biomass is a renewable source of energy. Technical Sciences. September 30, 2013, Vol. 3, pp. 211–220.

2. Tesfaldet Gebreegziabher, Adetoyese Olajire Oyedun, Ho Ting Luk, Tsz Ying Gene Lam, Yu Zhang, Chi Wai Hui. Design and optimization of a biomass power plant. Chemical Engineering Research and Design. s.l. : Elsevier B.V, 2014, pp. 1412–1427.

3. Tesfaldet Gebreegziabher, Adetoyese Olajire Oyedun, Chi Wai Hui**.** Optimum biomass drying for combustion - A modeling approach. Energy. s.l. : Elsevier Ltd., March 2, 2013, pp. 67 - 73.

4. Ho Ting Luk, Tsz Ying Lam, Adetoyese Olajire Oyedun, Tesfaldet Gebreegziabher, Chi Wai Hui**.** Drying of biomass for power generation: a case study on power generation from empty fruit bunch. Energy. s.l. : Elsevier Ltd., October 12, 2013, pp. 205 - 215.

5. Ming Liu, Xuwei Zhang, Xiaoqu Han, Gen Li, Junjie Yan**.** Using pre-drying technology to improve the exergetic efficiency of the bioenergy utilization process with combustion: A case study of a power plant. Applied Thermal Engineering. s.l. : Elsevier Ltd., September 1, 2017, pp. 1416 - 1426.

6. Peter, McKendry**.** Energy production from biomass (part 1): overview of biomass. Bio resource Technology. s.l. : Elsevier Science Ltd, 2002, pp. 37–46.

7. Tawfeeq, W. Mohammed**.** Biomass Energy. October 2021, pp. 1 - 9.

8. A. , K. Kurchania**.** Biomass Energy. India : s.n., 2012, 2, pp. 91 - 122.

9. Abdeen, Mustafa Omer **.** Biomass energy resources utilisation and waste management. Journal of Agricultural Biotechnology and Sustainable Development. October 2011, Vol. 3(8), pp. 149 -170.

10. Dr. Sa'ib , Abbas Hamid **.** Biomass Energy. Renewable Energy. 2018-2019, pp. 1 - 21.

11. Federal Democratic Republic of Ethiopia Ministry, of Finance**.** Investment Opportunities In Ethiopian Sugar Industry. April 2019, pp. 1 - 63.

12. Amornrat Kaewpradap, Wasan Yoksenakul and Sumrerng Jugjai**.** Effects of Moisture Content in Simulated Sugarcanebagasse by Equilibrium Analysis. the 4th TSME international conference on mechanical engineering. October 2013, pp. 16-18 .

13. Han Song, Fredrik Starfelt, Lilia Daianova, Jinyue Yan**.** influence of drying process on the biomass based polygeneration systemof bioethanol, power and heat. Applied Energy. s.l. : Elseveir Ltd, March 5, 2011, pp. 32 - 37.

14. E., Khalil**.** Steam power plants. Thermal Engineering in Power Systems. s.l. : WIT Press, 2008, Vol. 42, 4, pp. 100 - 139.

15. R.Jyothu.Naik, B.L.V.S.Gupta, G.S.Sharma**.** Exergy Analysis of 4.5mw Biomass Based Steam Power Plant. Journal of Humanities and Social Science. July - August 2012, Vol. 1, pp. 01-04.

16. Paul , Breeze**.** Biomass-Based Power generation. power generation technologies. s.l. : Elsevier Ltd, 2019.

17. Pavel, Kovařík**.** Drying of biomass with high water content. prague : s.n., January 2017, pp. 1 - 79.

18. Liu Jin, Tesfaldet Gebreegziabher, Zhang Yu, Adetoyese Olajire Oyedun, Zhu Yi, Wang Maojian, Chi Wai Hui**.** Modeling and optimization of microalgae drying for power generation. The 6th International Conference on Applied Energy. s.l. : Elsevier Ltd, 2014, pp. 168 – 171.

19. Angelo Del Giudice, Andrea Acampora , Enrico Santangelo , Luigi Pari , Simone Bergonzoli , Ettore Guerriero , Francesco Petracchini , Marco Torre , Valerio Paolini and Francesco Gallucci**.** Wood Chip Drying through the Using of a Mobile Rotary Dryer. Energies. April 26, 2019, pp. 1 - 16.

20. Hanning Li, Qun Chen, Xiaohui Zhang, Karen N Finney, Vida N Sharifi, Jim Swithenbank**.** evaluation of a biomass drying process using waste heat from process industries: a case study. pp. 1 - 16.

21. Peter, McKendry**.** Energy production from biomass (part 2): conversion technologies. Bioresource Technology. s.l. : Elsevier Science Ltd, 2002, pp. 47–54.

22. T, Gebregerges**.** “B ioenergy”(2012).

23. Daniele, Dell' Antonia**.** Biomass combustion Overview of Key Technologies - Benchmarking and Potentials. "Smart energy - Network of Excellence". 2013. pp. 1 - 70.

24. Scott Q. Turn, Bryan M. Jenkins, Lee A Jakeway, Linda G. Blevins, Robert B. Williams, Gary Ruben Stein, Charles M. Kinoshita**.** Test results from sugar cane sugarcanebagasse and high fiber cane co - fired with fossil fuels. Biomass and Bioenergy. s.l. : Elsevier Ltd, February 20, 2006, pp. 565 - 574.

25. Eva Thorin, Jan Sandberg, and Jinyue Yan**.** Combined Heat and Power. Clean Energy Systems. s.l. : John Wiley & Sons, Ltd, 2015, pp. 1 - 11.

26. Sustainable energy authority , of Ireland**.** Biomass Boilers - Technology Guide. March 2019. pp. 1 - 44.

27. Ryo, Nakakido**.** Commercialization possibilities of Small scale biomass power plant in Japan. Stockholm : s.n., 2016.

28. M. Valix, S. Katyal, W.H Cheung**.** combustion of thermochemically torrefied sugar cane sugarcanebagasse. Bioresource Technology. s.l. : Elsevier Ltd, october 21, 2016, pp. 202 - 209.

29. Eyerusalem, Birru**.** sugar cane industry overview and energy efficiency considerations. Stockholm, sweden  : s.n., March 2016. pp. 1 - 61.

30. Samuel, Oluwaseun Ogunrinde**.** Process concepts for conversion of biofuels residue to value added product. Manchester, United Kingdom : s.n., 2013. pp. 1 - 170.

31. USDA, Staff**.** Ethiopia Aims to Become One of the World’s Top 10 Sugar Producers. USDA Foreign Agricutural Service , Global Agricultural Information Network. Addis Ababa : s.n., 2015. pp. 1 - 8.

32. Sushil, Kumar**.** India’s Development Cooperation with Ethiopia in Sugar Production: An Assessment. Research and Information System for Developing Countries . New Delhi : s.n., August 2015. pp. 1 - 35.

33. Ji Gao, Aiping Zhang, Shu Kee Lam , Xuesong Zhang, Allison M. Thomson, Erda Lin , Kejun Jiang , Leon E. Clarke, James A. Edmonds , Page G. Kyle , Sha Yu , Yuyu Zhou and Sheng Zhou**.** An integrated assessment of the potential of agricultural and forestry residues for energy production in China. Bioenergy. s.l. : John Wiley & Sons Ltd, August 3, 2015.

34. Fateme Mohammadi, Anne Roedl, Mohammad Ali Abdoli, Majid Amidpour**.** Life cycle assesment (LCA) of the energetic use of sugarcanebagasse in iranian sugar industry. Renewable Energy. s.l. : Elsevier Ltd, June 27, 2019, pp. 1870 - 1882.

35. Kedir , Hussen Safeno**.** Value Chain Analysis Focusing on Organizational Production Process: The Case of Wonji/Shoa Sugar Factory. jimma, Ethiopia : s.n., June 2013. pp. 1 - 93.

36. Combustion air calculation. Combustion Technology. pp. 1 - 9.

37. Eric, M. Goodger**.** Fuels and combustion. pp. 1 - 19.

38. K., V. NARAYANAN**.** Chemical Engineering Thermodynamics. 2nd. Newdelhi : PHI Learning Private Limited, 2013. pp. 1 - 636.

39. G. Case , and E. State**.** Thermal Analysis of a Small-Scale Municipal Solid Waste-Fired Steam Generator : Case Study of Enugu State. Nigeria : s.n., December 2015.

40. Marc, Cortina**.** flue gas condenser for biomass boilers. Barcelona, spain : s.n., March 2006. pp. 1 - 76. ISSN.

41. M. T. Cheng and T. H. Zeng**.** Calculation and analysis of acid dew-point temperature in coal-fired boiler gas. 2015, pp. 614 - 617.

42. J. M. Smith, H. C. Van Ness, M. M. Abbott**.** Introduction to Chemical Engineering Thermodynamics. 6th. s.l. : McGraw-Hill, 2001. pp. 1 - 749.

43. Thermodynamics of Combustion. pp.205-235.(2011).

44. F. P. Estimation, W. Fuels, and R. D. Fuel**.** Predicting Dewpoints of Acid Gases. February 1981, 2017.

45. S. S. Hegde and B. R. Bhat, “Biomass waste-derived porous graphitic carbon for high-performance supercapacitors,” *J. Energy Storage*, vol. 76, p. 109818, Jan. 2024, doi: 10.1016/j.est.2023.109818.

46. F. J. Mascarenhas, S. S. Hegde, and B. R. Bhat, “Supercapattery: An Electrochemical Energy Storage Device,” in *Sustainable Materials for Electrochemical Capacitors*, 1st ed., Inamuddin, T. Altalhi, and S. M. Adnan, Eds., Wiley, 2023, pp. 279–290. doi: 10.1002/9781394167104.ch11.

47. J. Koliyoor, S. S. Hegde, Ismayil, and B. R. Bhat, “Exploring the microstructural properties of hydroxypropyl‐methylcellulose‐based solid polymer electrolytes: a promising candidate for flexible electrical double‐layer capacitor,” *Polym. Int.*, vol. 74, no. 11, pp. 965–980, Nov. 2025, doi: 10.1002/pi.70001.

48. S. S. Hegde and B. R. Bhat, “Solid Waste‐Derived Carbon Materials for Electrochemical Capacitors,” in *Sustainable Materials for Electrochemical Capacitors*, 1st ed., Inamuddin, T. Altalhi, and S. M. Adnan, Eds., Wiley, 2023, pp. 19–31. doi: 10.1002/9781394167104.ch2.

49. S. S. Hegde and B. R. Bhat, “Sustainable energy storage: *Mangifera indica* leaf waste-derived activated carbon for long-life, high-performance supercapacitors,” *RSC Adv.*, vol. 14, no. 12, pp. 8028–8038, 2024, doi: 10.1039/D3RA08910J.

50. S. Subraya Hegde and B. Ramachandra Bhat, “Impact of electrolyte concentration on electrochemical performance of Cocos nucifera Waste-Derived High-Surface carbon for green energy storage,” *Fuel*, vol. 371, p. 131999, Sept. 2024, doi: 10.1016/j.fuel.2024.131999.

51. B. A. G, R. S. Bhat, S. S. Hegde, and B. R. Bhat, “Electrochemical determination of ascorbic acid using carbon paste electrode modified with cobalt oxide nanoparticles,” *Electrochimica Acta*, vol. 542, p. 147526, Dec. 2025, doi: 10.1016/j.electacta.2025.147526.

52. S. S. Hegde *et al.*, “A novel and ultrasensitive high-surface porous carbon-based electrochemical biosensor for early detection of dengue virus,” *Biosens. Bioelectron. X*, vol. 20, p. 100525, Oct. 2024, doi: 10.1016/j.biosx.2024.100525.
